# An allosteric pan-TEAD inhibitor blocks oncogenic YAP/TAZ signaling and overcomes KRAS G12C inhibitor resistance

**DOI:** 10.1038/s43018-023-00577-0

**Published:** 2023-06-05

**Authors:** Thijs J. Hagenbeek, Jason R. Zbieg, Marc Hafner, Rana Mroue, Jennifer A. Lacap, Nicole M. Sodir, Cameron L. Noland, Shervin Afghani, Ayush Kishore, Kamakoti P. Bhat, Xiaosai Yao, Stephen Schmidt, Saundra Clausen, Micah Steffek, Wendy Lee, Paul Beroza, Scott Martin, Eva Lin, Rina Fong, Paola Di Lello, Marta H. Kubala, Michelle N.-Y. Yang, Jeffrey T. Lau, Emily Chan, Alfonso Arrazate, Le An, Elizabeth Levy, Maria N. Lorenzo, Ho-June Lee, Trang H. Pham, Zora Modrusan, Richard Zang, Yi-Chen Chen, Michal Kabza, Musaddeque Ahmed, Jason Li, Matthew T. Chang, Danilo Maddalo, Marie Evangelista, Xin Ye, James J. Crawford, Anwesha Dey

**Affiliations:** 1 Department of Discovery Oncology, Genentech, California, CA USA; 2 Department of Discovery Chemistry, Genentech, California, CA USA; 3 Department of Oncology Bioinformatics, Genentech, California, CA USA; 4 Department of Translational Oncology, Genentech, California, CA USA; 5 Department of Structural Biology, Genentech, California, CA USA; 6 Department of Biochemical and Cellular Pharmacology, Genentech, California, CA USA; 7 Department of Small Molecule Pharmaceutical Sciences, Genentech, California, CA USA; 8 Department of Protein Chemistry, Genentech, California, CA USA; 9 Department of Microchemistry, Proteomics and Lipidomics, Genentech, California, CA USA; 10 Department of Drug Metabolism and Pharmacokinetics, Genentech, California, CA USA; 11 Roche Polska, Warsaw, Poland; 12 Roche Canada, Mississauga, Ontario Canada

**Keywords:** Targeted therapies, Cancer therapeutic resistance, Drug development, Cancer

## Abstract

The Hippo pathway is a key growth control pathway that is conserved across species. The downstream effectors of the Hippo pathway, YAP (Yes-associated protein) and TAZ (transcriptional coactivator with PDZ-binding motif), are frequently activated in cancers to drive proliferation and survival. Based on the premise that sustained interactions between YAP/TAZ and TEADs (transcriptional enhanced associate domain) are central to their transcriptional activities, we discovered a potent small-molecule inhibitor (SMI), GNE-7883, that allosterically blocks the interactions between YAP/TAZ and all human TEAD paralogs through binding to the TEAD lipid pocket. GNE-7883 effectively reduces chromatin accessibility specifically at TEAD motifs, suppresses cell proliferation in a variety of cell line models and achieves strong antitumor efficacy in vivo. Furthermore, we uncovered that GNE-7883 effectively overcomes both intrinsic and acquired resistance to KRAS (Kirsten rat sarcoma viral oncogene homolog) G12C inhibitors in diverse preclinical models through the inhibition of YAP/TAZ activation. Taken together, this work demonstrates the activities of TEAD SMIs in YAP/TAZ-dependent cancers and highlights their potential broad applications in precision oncology and therapy resistance.

## Abstract (web summary)

Hagenbeek et al. identify a small-molecule pan-TEAD inhibitor that blocks the interaction between YAP/TAZ and TEAD proteins. They demonstrate that treatment with the inhibitor leads to antitumor activity and can synergize with KRAS G12C inhibition.

## Main

The Hippo signaling pathway controls multiple cellular functions, including proliferation, survival and differentiation^1,2^. Initial characterization of the pathway in *Drosophila* and mammals revealed a conserved linear serine/threonine kinase cascade: MST1 (Ste20-like protein kinase 1) and MST2 (Ste20-like protein kinase 2) phosphorylate and activate LATS1 (large tumor suppressor 1) and LATS2 (large tumor suppressor 2), which in turn phosphorylate the key downstream transcription co-activators YAP (Yes-associated protein) and TAZ (transcriptional coactivator with PDZ-binding motif) and lead to their cytosolic retention and eventual degradation. When YAP/TAZ are not phosphorylated, they are translocated into the nucleus, where they bind to the TEAD family of transcription factors to turn on a transcriptional program that drives cell proliferation and survival^1–4^. Since the initial discovery of the canonical kinase components of the Hippo pathway, a wide variety of growth-promoting upstream signals have been reported to activate YAP/TAZ through negative regulation of the Hippo pathway, as well as through Hippo-independent mechanisms^3–10^. These have been covered extensively in recent reviews^1–6^.

YAP/TAZ have emerged as prominent drivers of human cancers^4,5,11–14^. Constitutively active YAP/TAZ have been shown to drive tumorigenesis in the liver and mammary gland in genetically engineered mouse models or through hydrodynamic tail vein injections^15–18^. YAP/TAZ can be activated in human tumors through overexpression or amplification or the loss of upstream negative regulators, such as NF2 (ref. ^12^), or nongenetically by a variety of upstream signals including those mentioned above. For instance, the stability and expression of YAP/TAZ have been shown to be modulated by the RAS family of small GTPases, and the transcriptional program of YAP/TAZ plays pivotal roles in driving such oncogenic programs^4^. YAP activation can compensate for KRAS inhibition in KRAS-driven murine models of cancer and enable KRAS-independent tumor growth^19,20^. Similarly, YAP/TAZ are known to drive tumor proliferation and resistance in response to a variety of targeted therapies, including EGFR (epidermal growth factor receptor), ALK (anaplastic lymphoma kinase), MEK (mitogen-activated protein kinase kinase) and CDK4/6 (cyclin-dependent kinase 4/6) inhibitors^19–26^. For these reasons, pharmacological inhibitors that can block the transcriptional program downstream of YAP/TAZ hold great promise in cancer therapies.

Activation of the transcriptional program downstream of YAP/TAZ is orchestrated through their interaction with the TEAD family (TEAD1­–4) of transcription factors^27,28^. In hydrodynamic tail vein injection mouse models, disruption of YAP/TAZ binding to TEAD was found to prevent cancer onset, and small interfering/small hairpin RNA-mediated repression of YAP, TAZ and TEAD disrupted YAP/TAZ-dependent human cancer cell line growth both in vitro and in vivo^15,29^. Although YAP/TAZ lack apparent druggable pockets, the discovery of the lipid pocket on TEADs^30^ has led to the development and evaluation of small-molecule inhibitors (SMIs) of TEADs, with varying levels of success^1,31–35^. Of particular note, the functional redundancy of the four human TEAD paralogs represents a major hurdle in such drug discovery processes. Furthermore, earlier-generated TEAD lipid pocket binders were ineffective in blocking YAP/TAZ binding to TEADs, showing limited activity and antiproliferative effects^1^. In this article, we report the discovery of a potent SMI that reversibly binds to all four human TEAD paralogs and allosterically blocks YAP/TAZ binding. We demonstrate that this molecule exhibits broad activity in cancer cells harboring genetic alterations in the Hippo pathway. Moreover, we demonstrate the use of this pan-TEAD SMI as a strategy to overcome resistance to sotorasib (an inhibitor of the KRAS variant with a p.Gly12Cys alteration; hereafter, KRAS G12C). This highlights its broad potential application in both YAP/TAZ-dependent tumors and in combination with other targeted cancer therapies.

## Results

### Discovery of an SMI series targeting the TEAD lipid pocket

**Fig. 1:**
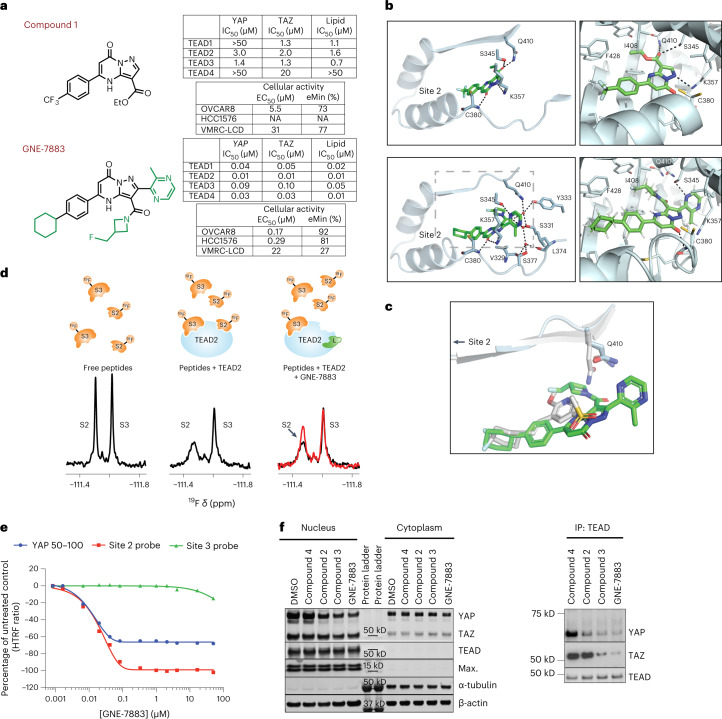
Discovery and characterization of the potent pan-TEAD inhibitor GNE-7883. **a**, Chemical structures and key biochemical and cellular activity data for compound 1 and GNE-7883. **b**, Crystal structure of compound 1 (top row) and GNE-7883 (bottom row) bound in the TEAD2 lipid pocket. Site 2 is shown for reference. The compounds are shown in green stick representation. **c**, Overlay of the crystal structures of GNE-7883 bound to TEAD2 and a compound known to bind in the lipid pocket but not inhibit YAP/TAZ binding (Protein Data Bank accession 6UYC, colored gray). **d**, Left, ^19^F NMR spectrum of fluorinated peptides S2 and S3 at 20 μM. Middle, ^19^F NMR spectrum of fluorinated peptides S2 and S3 at 20 μM in the presence of 10 μM TEAD2. The ^19^F NMR signals for the free S2 and S3 peptides are reduced upon binding to TEAD2. Right, overlay between the ^19^F NMR spectra of 20 μM S2 and S3 in the presence of 10 μM TEAD2 (black trace) and after the addition of 55 μM GNE-7883 (red trace). **e**, Dose–response curve showing displacement of the site 2 probe by GNE-7883, confirming allosteric perturbation of TEAD2 site 2. Under the same assay conditions, YAP 50–100 displacement plateaus at 60%, while no perturbation of the site 3 probe is observed with GNE-7883. The experiment was performed once with two technical replicates. **f**, Nuclear and cytosolic fractionation (left) of YAP-amplified OVCAR-8 cells treated with 3 μM TEAD SMI or dimethyl sulfoxide (DMSO). In parallel, reciprocal immunoblotting of YAP and TAZ was conducted following immunoprecipitation with a pan-TEAD antibody (right). The experiments were repeated twice with consistent results. Source data

Given the premise that YAP/TAZ dissociation from TEAD is central to inactivation of the pro-tumor transcriptional program downstream of YAP/TAZ, we sought to identify pan-TEAD inhibitors that block the association of YAP/TAZ with TEADs. We conducted a high-throughput screen of >2 million compounds using a TEAD3–YAP time-resolved fluorescence resonance energy transfer (TR-FRET) assay (Supplementary Table 1). A total of 1,680 small-molecule hits exhibited ≥20% inhibition of the TR-FRET signal, representing a 0.076% hit rate. Of the compounds selected for half-maximum inhibitory concentration (IC_50_) follow up, 353 were classified as active based on an activity of <5 μM, representing a 21% confirmation rate. Of these, compound 1 (Fig. 1a) was of particular interest based on its TEAD3–YAP IC_50_ of 1.4 μM, attractive physiochemical properties and drug-like molecular weight. When this compound was tested against the interactions between other TEAD paralogs and both YAP and TAZ, we noticed marked differences, particularly with TEAD4 (>50 μM). While there is significant homology between TEADs 1–4 at the recognized protein–protein interaction (PPI) interfaces, there is more apparent divergence in the lipid-binding pocket. Indeed, using a lipid displacement assay (Fig. 1a), we identified the same selectivity trend for compound 1 against TEADs 1–4, indicating that although compound 1 was identified as a PPI blocker it still binds within the TEAD lipid pocket and blocks the PPI via an unanticipated allosteric mechanism.

**Extended Data Fig. 1:**
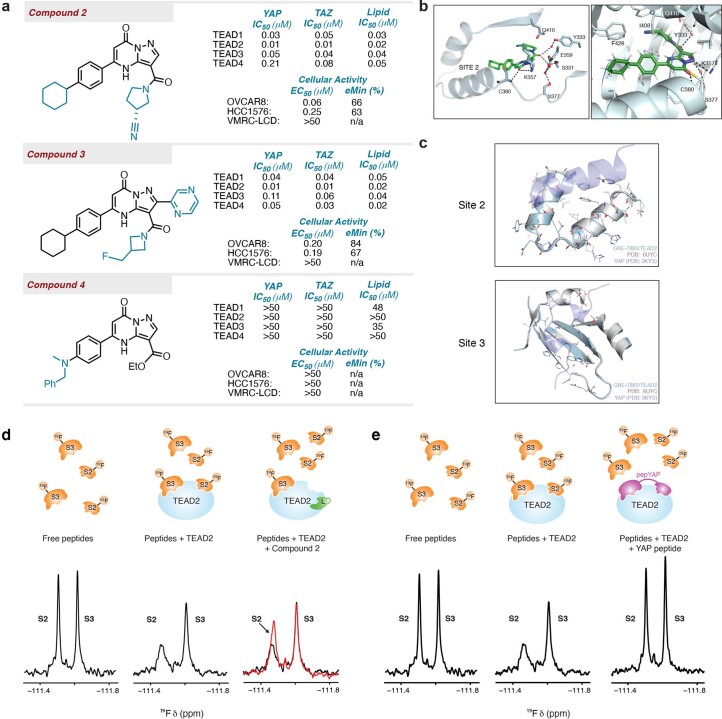
Discovery of small molecule inhibitors blocking TEAD interaction with YAP/TAZ. **(a)** Chemical structures and key biochemical and cellular activity data for Compounds 2, 3, and 4. **(b)** Crystal structure of Compound 2 bound in the TEAD2 lipid pocket. Site 2 is shown for reference. Compound is shown in green stick representation. **(c)** Overlay of Sites 2 and 3 for the structures of GNE-7883 bound to TEAD2 (blue) and a non-allosteric lipid pocket compound bound to TEAD2 (PDB 6UYC, grey). The YAP Site 2 helix or Site 3 omega loop from PDB 3KYS is overlaid in transparent purple for reference. **(d)** (Left) ^19^F NMR spectrum of fluorinated peptides S2 and S3 at and (Middle) in the presence of TEAD2. The ^19^F NMR signals for the free S2 and S3 peptides are reduced upon binding to TEAD2. (Right) Overlay of the ^19^F NMR spectra of S2 and S3 in the presence of TEAD2 (black trace) and after addition of Compound 2 (red trace). **(e)** (Left) ^19^F NMR spectrum of fluorinated peptides S2 and S3 at 20 μM. (Middle) ^19^F NMR spectrum of peptides S2 and S3 at 20 μM in the presence of 10 μM TEAD. (Right) ^19^F NMR spectrum of peptide S2 and S3 at 20 μM in the presence of 10 μM TEAD2-YAP and 55 μM pepYAP. Source data

Based on this finding, we set out to elucidate the structural basis of such allosteric inhibition and to discover more potent ligands with pan-TEAD activity. We optimized van der Waals interactions in the lipophilic portion of the pocket by replacing the trifluoromethyl with a cyclohexyl and added a cyanopyrrolidine amide in place of the ethyl ester (compound 2; Extended Data Fig. 1a). This not only improved lipid pocket binding but also resulted in significantly improved pan-TEAD PPI activity against both YAP and TAZ (Extended Data Fig. 1a). Furthermore, this compound showed promising antiproliferative activity in YAP-amplified OVCAR-8 and NF2-null HCC1576 cell lines, while being inactive in a control cell line (VMRC-LCD) (Extended Data Fig. 1a). We obtained co-crystal structures of compounds 1 and 2 bound to TEAD2 (Fig. 1b, Extended Data Fig. 1b and Supplementary Table 2). We found that compound 1 makes hydrogen bonding interactions with Lys357, Gln410, Ser345 and the main-chain amide of Cys380. In contrast, compound 2 loses the interactions with Gln410 and Ser345 and picks up a series of water-mediated interactions with Tyr333, Glu359, Ser331 and Ser377, which helps to drive an increase in potency.

With the observed cellular phenotype and structural information, we set out to improve on compound 2 with a particular focus on pan-TEAD potency. Notably, targeting Ser345 (TEAD2) was of high interest given the potential to form a productive hydrogen bond with a conserved hydroxyl motif across all TEAD paralogs. As seen in the co-crystal structures of compound 2, Ser345 (TEAD2) sits in an unoccupied hydrophilic region near the entrance of the ligand-binding pocket. We appended polar groups, such as a pyrazine ring, from the biaryl core of compound 2. We found that this change enabled the formation of a productive hydrogen bond with the relevant side chain hydroxyl group of Ser345 (TEAD2). Introducing these new interactions within this polar vestibule of the pocket led to potency gains across all TEAD paralogs. Replacement of the cyanopyrrolidine with a 4-fluoromethyl azetidine to balance the overall polarity led us to compound 3 (Extended Data Fig. 1a). Lastly, appending a methyl group on the pyrazine heterocycle in compound 3 resulted in GNE-7883, which showed substantial biochemical potency gain against TEAD4 and increased antiproliferative effects (Fig. 1a). Mechanistically, a co-crystal structure of GNE-7883 bound to TEAD2 confirmed that the methylpyrazine achieves the interaction with Ser345 and expands the water-mediated interaction network to include Ser377 and the main-chain carbonyls of Leu345 and Val329 (Fig. 1b,c).

### GNE-7883 allosterically inhibits the binding of YAP/TAZ to TEADs

We were surprised to see no major disturbances of the PPI interfaces, broadly constituting site 2 and site 3 (refs. ^36,37^) in the co-crystal structures, compared with known structures of palmitoylated TEAD2 or TEAD2 bound to a ligand that does not affect YAP binding (Extended Data Fig. 1c) (ref. ^36^). Instead, this new SMI series binds between the first and second TEAD helices that form site 2 and displaces Gln410—a residue proximal to site 2—within the lipid pocket (Fig. 1b). This led us to hypothesize that this chemical series may create tension between the two helices, altering the conformational landscape of site 2 in solution and leading to PPI inhibition.

To confirm this allosteric modulation of site 2, we conducted a set of complementary fluorine NMR and biochemical competition assays. We incubated S2 and S3—two ^19^F-labeled peptides encompassing the YAP sequences that bind TEAD at site 2 and site 3, respectively—with TEAD2 and then added either compound 2 or GNE-7883 to monitor the displacement of the fluorinated peptides. First, we incubated S2 and S3 with substoichiometric amounts of TEAD2 and, upon binding of the peptides to TEAD2, we observed a decrease in the intensity of the ^19^F signals for the free peptides in the one-dimensional ^19^F NMR spectrum (Fig. 1d and Extended Data Fig. 1d). The ^19^F signals for the bound forms of both peptides were broadened beyond detection due to a change in the relaxation properties of the peptides when bound to a large protein such as TEAD2. Upon the addition of compound 2 or GNE-7883 to samples containing S2, S3 and TEAD2, we observed a partial increase in the ^19^F signal of free peptide S2, whereas the ^19^F NMR signal of free peptide S3 remained virtually unchanged (Fig. 1d and Extended Data Fig. 1d). This observation suggests that not only do both compound 2 and GNE-7883 bind in the lipid pocket, but they also allosterically perturb the binding of peptide S2 at the site 2 pocket. As a control, we also added YAP peptide encompassing both site 2 and site 3 binding regions (amino acids 60–100; pepYAP) to a sample containing S2, S3 and TEAD2. As expected, we observed an increase in the signals of free S2 and S3, indicating that both peptides were displaced upon the binding of pepYAP to TEAD2 (Extended Data Fig. 1e).

As an orthogonal approach, we performed competitive homogeneous time-resolved fluorescence (HTRF) assays using biochemical probes. These were based on either: (1) a Vgll1 peptide, a transcriptional coactivator that binds to TEAD at site 2 (site 2 probe); or (2) peptide 17, which binds to site 3 (site 3 probe) (refs. ^38,39^). These experiments clearly showed that our compounds competed with Vgll1 binding, but not peptide 17, and thus blocked the binding of YAP/TAZ via site 2 (Fig. 1e). Taken together, our data provide strong evidence for allosteric blockade of YAP/TAZ binding at site 2.

### GNE-7883 displaces YAP/TAZ and suppress their activities

**Fig. 2:**
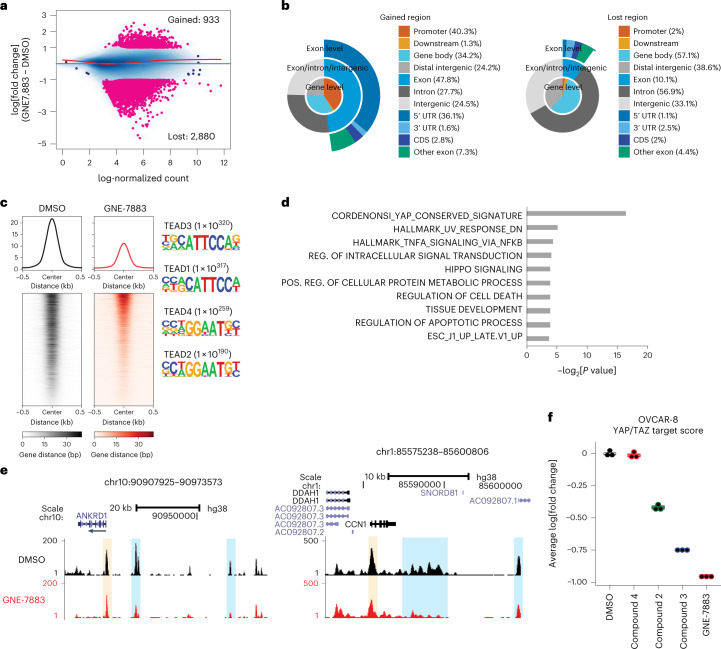
GNE-7883 specifically decreases chromatin accessibility at TEAD motifs and YAP/TAZ target genes. **a**, Differential analysis of ATAC-seq of OVCAR-8 cells treated with dimethyl sulfoxide (DMSO) or GNE-7883 for 48 h (*n* = 3 independently treated cell cultures). The pink points are significantly altered regions. The red line is a fitted Lowess curve through all of the regions. **b**, Genomic annotations of the differential ATAC-seq peaks by ChIPAnnotate. CDS, coding sequence; UTR, untranslated region. **c**, deepTools visualization of lost ATAC-seq peaks and motif enrichment analysis. **d**, Lost peaks were assigned to genes using known distal enhancer–gene target links in Poly-Enrich and the ten most significant pathways were computed by gene set enrichment. *P* values was derived from one-sided binomial test. **e**, UCSC genome browser view of ATAC-seq at known targets of TEAD, ANKRD1 (ankyrin repeat domain 1) and CCN1 (cellular communication network factor 1). Promoter regions are shaded in yellow and enhancer regions are shaded in blue. The experiments were repeated twice with consistent results. **f**, Box plots showing aggregated expression level changes of the YAP/TAZ target genes, as measured by messenger RNA-seq in OVCAR-8 cells under the indicated treatments for 48 h (*n* = 3 independently treated cell cultures). The data are presented as medians ± 25%. The lower and upper whiskers extend from the hinge to the smallest or largest values. Source data

**Extended Data Fig. 2:**
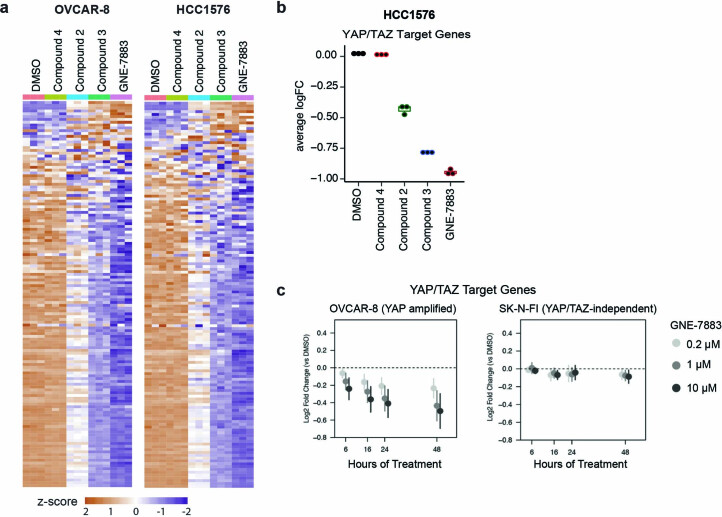
TEAD SMIs selectively modulate YAP/TAZ target genes. **a)** Heatmap showing expression of the YAP/TAZ target genes measured by mRNA-sequencing in OVCAR-8 and HCC1576 cells treated with the indicated compounds (n = 3 independently treated cell cultures). **(b)** Box plot showing aggregated expression changes of the YAP/TAZ target genes in HCC1576 cells treated with the indicated compounds (n = 3 indepedently treated cell culture). Data are presented as median +/− 25%. The lower and upper whiskers extend from the hinge to the smallest or largest values. **(c)** Downregulation of YAP/TAZ target genes in a time- and concentration-dependent manner in YAP-amplified OVCAR-8 cells (left), but not in control NF2 wildtype, YAP/TAZ negative SK-N-FI cells (right). Error bars represent SD (n = 3 independently treated cell cultures). Source data

Consistent with the mechanism of action of these compounds, we found that they did not alter the nuclear and cytosolic localization of YAP, TAZ and TEAD but displaced YAP/TAZ from TEAD in a potency-dependent manner (Fig. 1f). Using assay for transposase-accessible chromatin with sequencing (ATAC-seq), we confirmed that TEAD SMI treatment resulted in specific remodeling of the chromatin in OVCAR-8 cells. Following a 48 h treatment with GNE-7883, we detected 933 gained, 2,880 lost and 133,029 unaltered regions (absolute log_2_[fold change] > 1 and false discovery rate (FDR) < 0.01; negative binomial test) (Fig. 2a). The lost regions were found predominantly at distal regions, whereas the gained regions were promoter centric (Fig. 2b). Systematic motif enrichment analysis identified TEAD-specific motifs in regions of reduced chromatin accessibility (Fig. 2c). Since the majority of the downregulated peak regions were distal, we assigned enhancer–gene targets using links derived from aggregated chromatin capture data by Poly-Enrich^40^. The most significant pathway corresponded to YAP/TAZ target genes, consistent with GNE-7883’s specificity in modulating YAP, TAZ and TEAD activity (Fig. 2d). As an example, we observed a prominent decrease in chromatin accessibility at the promoter and multiple enhancer regions of well-known YAP/TAZ targets, such as ANKRD1 (ankyrin repeat domain 1) and CCN1 (cellular communication network factor 1, also known as CYR61) (Fig. 2e). On the transcript level, we confirmed that this series of SMIs suppressed the expression of Hippo pathway target genes defined previously^29^ in multiple models, and the level of suppression improved according to the potency of the compounds (Fig. 2f and Extended Data Fig. 2a,b). Finally, in OVCAR-8 cells, but not in YAP/TAZ-nonexpressing SK-N-FI cells, GNE-7883 decreased the expression of YAP/TAZ target genes in time- and dose-dependent fashion (Extended Data Fig. 2c), confirming its specificity.

### GNE-7883 inhibits the growth of YAP/TAZ-dependent cancer cells

**Fig. 3:**
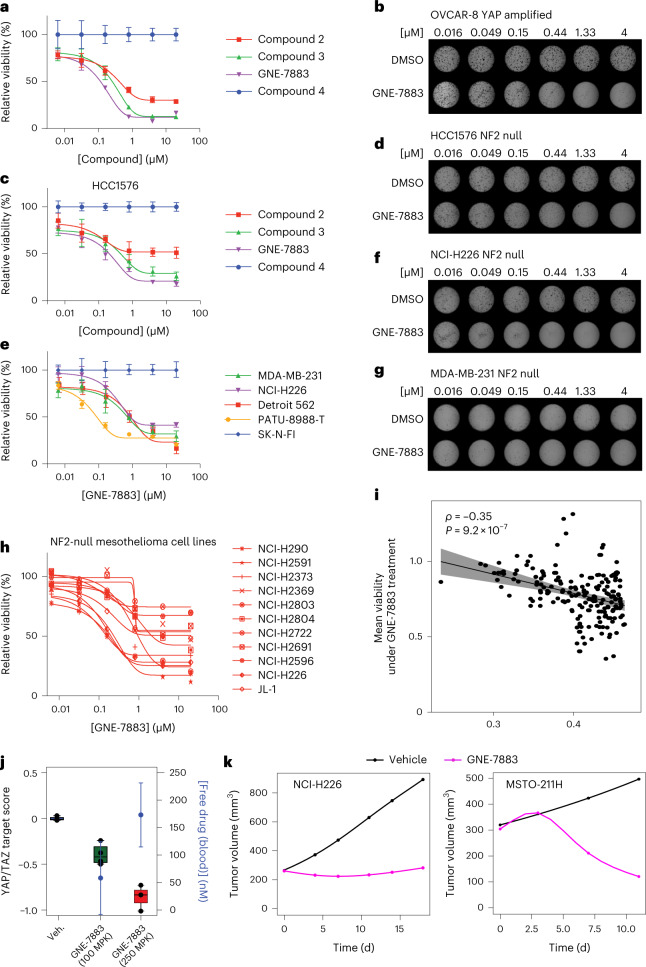
GNE-7883 inhibits the growth of YAP/TAZ-dependent cell lines in vitro and in vivo. **a**, Viability dose–response curves (means ± s.d.) of OVCAR-8 cells treated with TEAD SMIs (*n* = 10 independently treated cell cultures). **b**, Soft agar colony formation dose responses of OVCAR-8 cells treated with GNE-7883 versus dimethyl sulfoxide (DMSO) control. The experiment was performed twice with similar results. **c**, Viability dose–response curves (means ± s.d.) of HCC1576 cells treated with TEAD SMIs (*n* = 5 independently treated cell cultures). **d**, Soft agar colony formation dose responses of HCC1576 cells treated with GNE-7883 versus DMSO control. The experiment was performed twice with similar results. **e**, Viability dose–response curves (means ± s.d.) of YAP/TAZ-dependent OVCAR-8, HCC1576, MDA-MB-231 and NCI-H226 cells versus YAP/TAZ-independent SK-N-FI cells treated with GNE-7883 (*n* = 5 independently treated cell cultures per condition). **f**,**g**, Soft agar colony formation dose responses of NCI-H226 (**f**) and MDA-MB-231 (**g**) cells treated with GNE-7883 or DMSO control. The experiment was performed twice with similar results. **h**, Viability dose–response curves (means ± s.d.) of NF2-null mesothelioma cell lines treated with GNE-7883 (*n* = 5 independently treated cell cultures) **i**, Cell viability responses to GNE-7883 in 196 cell lines correlate (Spearman’s *ρ* = −0.35; *P* = 9.2 × 10^−7^) with baseline transcriptional YAP/TAZ target scores. The error band represents the 95% confidence interval. **j**, Pharmacodynamic analysis of GNE-7883, including unbound compound concentrations (means ± s.d.) in the blood and YAP/TAZ target scores of NCI-H226 xenograft tumors treated with GNE-7883 once daily for 4 days (*n* = 4 mice per group). The lower and upper hinges of the boxes correspond to the first and third quartiles (that is, the 25th and 75th percentiles). The lower and upper whiskers extend from the hinge to the smallest or largest values. MPK, milligrams per kilogram. **k**, In vivo efficacy study of mice bearing NCI-H226 (left; *n* = 10 mice per group) and MSTO-211H (right; *n* = 9 mice per group) xenograft tumors treated with GNE-7883 (magenta) at 250 mg kg^−1^ (4 d on 2 d off) or control vehicle (black) until the end of the treatment. Source data

**Extended Data Fig. 3:**
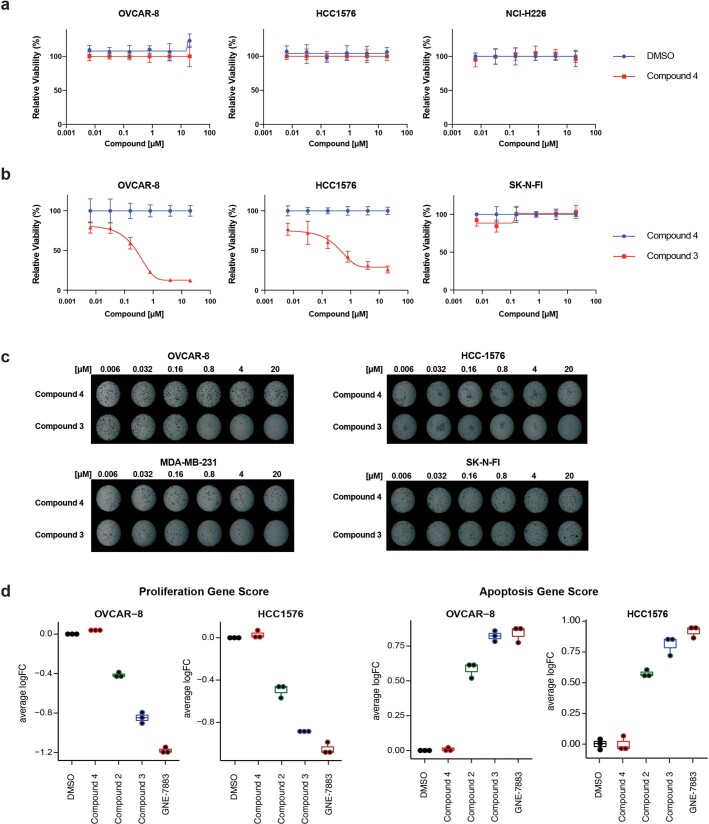
Compound 4 does not impact growth of YAP/TAZ-dependent cell lines *in vitro*. **(a)** Viability dose-response curves (mean±SD) of OVCAR-8 cells, HCC1576, and NCI-H226 cells treated with Compound 4 or DMSO. (n > 5 replicates per condition) **(b)** Viability dose-response curves (mean±SD) of OVCAR-8 cells, HCC1576 cells, versus YAP/TAZ-independent SK-N-FI cells treated with Compound 3 or Compound 4. (n = 5 independently treated cell cultures per condition) **(c)** Soft agar colony formation dose-response of OVCAR-8 cells, HCC1576 and MDA-MB-231 cells, versus YAP/TAZ-independent control SK-N-FI cells treated with Compound 3 or Compound 4. Experiments were repeated twice with similar results. **(d)** Box plot showing aggregated expression changes of proliferative and apoptosis genes measured by mRNA-sequencing in OVCAR-8 and HCC1576 cells treated with the indicated compounds (n = 3 independently treated cell cultures). Data are presented as median +/− 25%. The lower and upper whiskers extend from the hinge to the smallest or largest values. Source data

Next, we compared the antiproliferative activity of these TEAD SMIs in YAP/TAZ-dependent versus independent cell lines^29^. We used a structurally related small molecule, compound 4, as a negative control. Compound 4 does not bind TEADs, nor does it suppress TEAD binding with YAP/TAZ or expression of the YAP/TAZ targets (Fig. 1f and Extended Data Figs. 1a and 2a), and it did not impact the survival of YAP/TAZ-dependent cells (Extended Data Fig. 3a–c). In contrast, GNE-7883 showed a strong dose-dependent antiproliferative effect and improvement over compounds 2 and 3 in both OVCAR-8 and HCC1576 cells (Fig. 3a–d and Extended Data Fig. 3d). Next, we evaluated two additional NF2 (moesin-ezrin-radixin like tumor suppressor 2) null cell lines (MDA-MB-231 and NCI-H226) and two cell lines with Hippo pathway dysregulation (PA-TU-8988T and Detroit 562). These variant cell lines showed a similar dose-dependent response to GNE-7883 (Fig. 3e–g). In contrast, SK-N-FI cells did not show any sensitivity to GNE-7883 (Fig. 3e).

Next, we extended our characterization of GNE-7883 to additional cell line models. First, as NF2 deficiency was previously reported as a biomarker of YAP/TAZ dependency and is frequently found in mesothelioma^12,29,41–43^, we tested GNE-7883 across a panel of NF2-null mesothelioma cell lines and validated that these models are overall responsive to GNE-7883 (Fig. 3h). Second, given that YAP/TAZ can be activated by a wide variety of signals in addition to NF2, we investigated whether heightened YAP or TAZ transcriptional activity may correlate with GNE-7883 sensitivity across cell lines. We assessed the antigrowth activity of GNE-7883 across 196 NF2 wild-type cancer cell lines spanning various indications. Cell viability after GNE-7883 treatment was significantly anticorrelated with the baseline YAP/TAZ target score (Fig. 3i; Spearman’s *ρ* = −0.35; *P* = 9.2 × 10^−7^), supporting the notion that YAP/TAZ activity is associated with YAP/TAZ dependency and sensitivity to TEAD inhibition.

**Extended Data Fig. 4:**
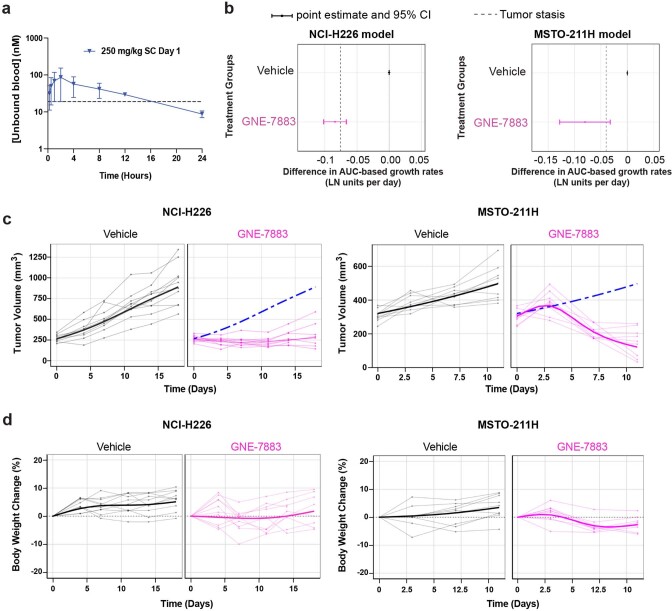
TEAD SMIs inhibit growth of YAP/TAZ-dependent cell lines *in vitro* and *in vivo*. **(a)** Pharmacodynamic analysis of GNE-7883 by measuring unbound compound concentration in blood (mean±SD). EC_50_ (unbound values derived from in vitro assays and corrected for 88.3% binding to 10% FCS) concentration is indicated by the dotted line. n = 4 mice per timepoint. **(b)** Growth contrast plot comparing growth rates (point estimate ± 95% confidence interval) of tumors treated with GNE-7883 at 250 mg/kg, 4 days on 2 days off (magenta) versus the vehicle control (black). n = 10 animals per group. **(c)** Fitted tumor volume estimates are shown alongside tumor measurement of individual animals over time. Dashed blue lines in the right plot represent the fitted tumor growth curve under vehicle treatment. n = 10 mice per group. **(d)** Percent changes in body of mice treated with indicated compounds over time. The fitted estimates are shown alongside with body weight measurement of individual animals. n = 10 mice per group. Source data

The strong activity of GNE-7883 in vitro prompted us to further assess its activity in vivo. The oral exposure of GNE-7883 was low (oral bioavailability = 6% at 25 mg kg^−1^), leading us to test an alternate route of administration via subcutaneous dosing. To measure YAP/TAZ target modulation by GNE-7883 in vivo, we treated the NCI-H226 xenograft model with either vehicle control or GNE-7883 at 100 or 250 mg kg^−1^ once daily for 4 d. Tumor tissue analysis showed a dose-dependent decrease of the YAP/TAZ target score in tumors treated with GNE-7883, as well as a dose-dependent increase in the blood concentration of the compound, suggesting a good correlation between pharmacokinetics and pharmacodynamics effects (Fig. 3j and Extended Data Fig. 4a). Next, we conducted efficacy studies in two widely used YAP/TAZ-dependent xenograft models: NCI-H226 and MSTO-211H^41^. GNE-7883 treatment achieved tumor stasis in the NCI-H226 model and resulted in tumor regression in the MSTO-211H model. The treatment was tolerated as no treatment-associated body weight loss was observed (Fig. 3k and Extended Data Fig. 4b–d).

### GNE-7883 overcomes resistance to KRAS G12C inhibition

**Extended Data Fig. 5:**
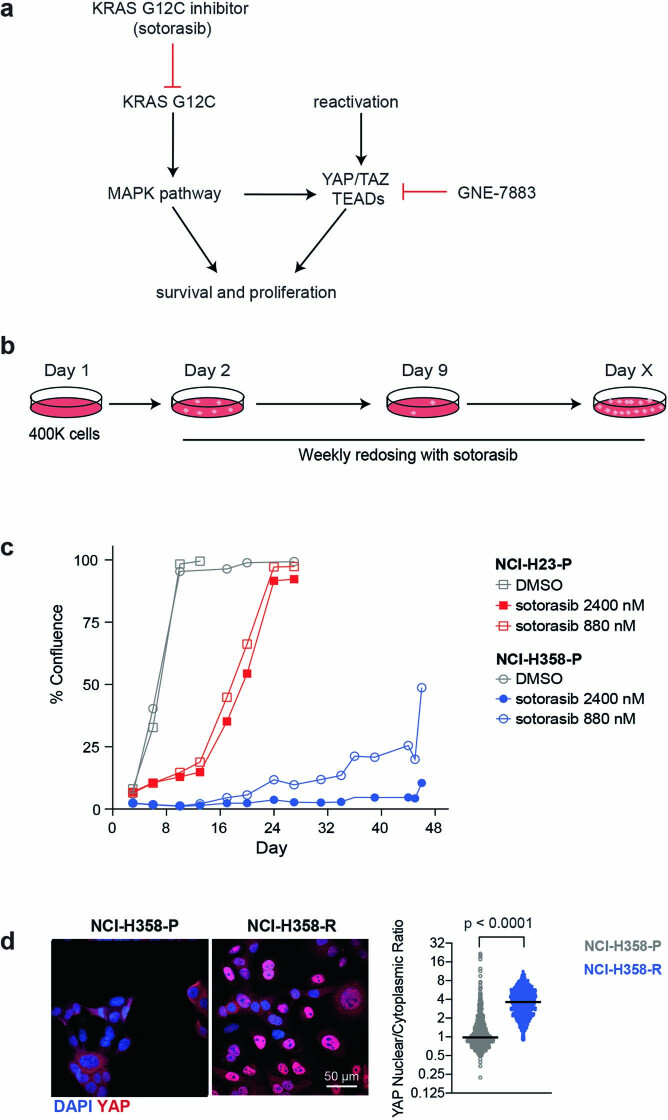
Development of sotorasib resistant lung cell line models. **(a)** Schematic showing cross talk between KRAS/MAPK signaling and YAP/TAZ activity and how GNE-7883 may overcome resistance to KRAS G12C inhibition by sotorasib. **(b)** Schematic showing resistance modeling KRAS G12C mutant lung cancer cell line models. **(c)** Graph of cell growth dynamics and adaptation of NCI-H23 and NCI-H358 cells through sotorasib treatment assessed as percent plate confluence using Incucyte. Experiments were repeated three times with similar results. **(d)** Immunofluorescence images showing YAP staining (red) in NCI-H358-P cells (left panel) versus in NCI-H358-R cells (right panel). The graph shows quantification of nuclear/cytoplasmic ratio of YAP (n = 801 cells for NCI-H358-P and n = 1494 cells for H358-R). p value was derived from two-sided Wilcoxon Rank Sum Test. The experiment was performed three times with similar results. Source data

As mentioned, YAP/TAZ constitute a robust resistance mechanism to a variety of targeted agents. We went on to explore the usage of GNE-7883 as a combination partner with SMIs targeting the most frequently mutated human oncogene, KRAS. YAP/TAZ and the transcriptional program downstream of the KRAS/MAPK (mitogen-activated protein kinase) pathway have been shown to cross-talk at multiple levels and converge on an overlapping set of target genes^20,29^. In KRAS G12D driven murine models of lung cancer, YAP activation may compensate for KRAS depletion and enable continued tumor growth upon KRAS G12D inhibition^19,20^. The recent Food and Drug Administration approval of the KRAS G12C-selective inhibitor sotorasib^44^ in KRAS G12C-mutant non-small cell lung cancers (NSCLCs) represented a milestone in treating KRAS-driven cancers. Nonetheless, the clinical response to sotorasib was limited to a fraction of patients with a median progression survival of 6.8 months from the initial clinical trial^45^. Among the patients who responded to KRAS G12C inhibitors but later acquired resistance, over one-third do not harbor any detectable putative, resistance-conferring, treatment-emergent genetic alterations, indicating that nongenetic bypass mechanisms play a crucial role in driving both intrinsic and adaptive resistance to sotorasib^46,47^. Accordingly, we evaluated whether the resistance to sotorasib could be overcome by inactivation of the YAP/TAZ–TEAD transcriptional program (Extended Data Fig. 5a).

**Fig. 4:**
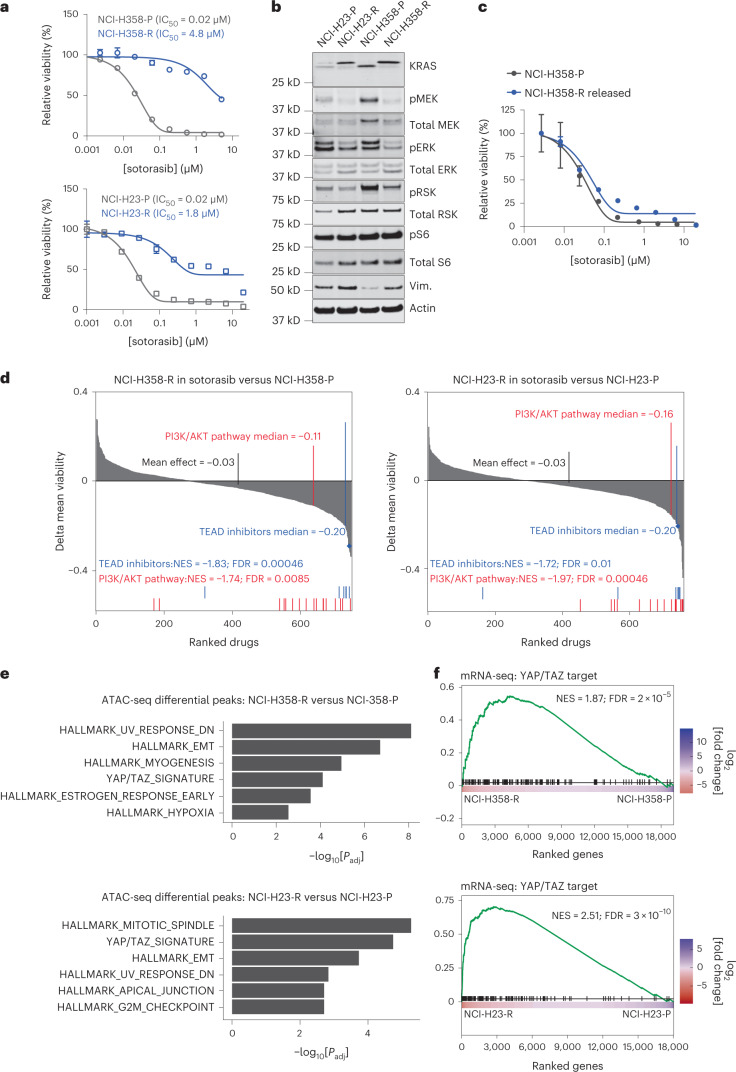
The YAP/TAZ transcriptional program is a prominent driver of KRAS G12C inhibitor resistance in lung cancer cells. **a**, Sotorasib viability dose–response curves (means ± s.d.) for NCI-H358-P and NCI-H358-R cells (top) and NCI-H23-P and NCI-H23-R cells (bottom) (*n* = 3 independently treated cell cultures per condition). **b**, Western blot analysis showing KRAS G12C alkylation and target engagement and key pathway nodes in untreated parental cells versus NCI-H23-R and NCI-H358-R cells maintained in sotorasib. The experiments were repeated four times with consistent results. Vim., vimentin. **c**, Sotorasib viability dose–response curves (means ± s.d.) for parental NCI-H358-P cells and NCI-H358-R cells passaged in drug-free media for 8 weeks (NCI-H358-R released) (*n* = 3 independently treated cell cultures per condition) **d**, Chemical genetic screens of 720 small molecules, assessing differences in the mean viability values between sotorasib-resistant NCI-H358-R (left) and NCI-H23-R (right) cells versus their corresponding parental lines. The most enriched drug classes are shown, along with the median delta mean viability, normalized enrichment score (NES) and corresponding FDR value. The blue diamonds represent GNE-7883. **e**, Pathway enrichment for genes associated with upregulated ATAC-seq peaks in sotorasib-resistant NCI-H358-R (top) and NCI-H23-R (bottom) cells compared with parental cells under acute sotorasib treatment. *P*_adj_, adjusted *P* value. **f**, GSEA plot showing the enrichment of YAP/TAZ target genes among differentially expressed genes in sotorasib-resistant NCI-H358-R (top) and NCI-H23-R (bottom) cells and their corresponding parental cells under acute sotorasib treatment for 24 h. For **d**–**f**, the *P* values were calculated using GSEA and adjusted using Benjamini–Hochberg procedure. Source data

To test this hypothesis, we derived sotorasib-resistant NCI-H23 and NCI-H358 KRAS G12C mutant lung cell line models by treating them with clinically relevant doses of sotorasib until resistant cells emerged (Extended Data Fig. 5b). These models elicited two different temporal dynamics of adaptation to sotorasib: it took several weeks for NCI-H358 cells to recover from substantial cell death and adapt, whereas NCI-H23 cells only underwent brief cytostasis, and outgrowths were apparent within 2 weeks (Extended Data Fig. 5c). In both resistance models (Fig. 4a), sotorasib retained its ability to alkylate KRAS G12C and suppress the MAPK pathway (Fig. 4b). Moreover, when we passaged sotorasib-resistant NCI-H358 cells in the absence of sotorasib (sotorasib release) for 8 weeks, we observed a clear reversal of the resistance phenotype (Fig. 4c). The reversal was not observed with NCI-H23 cells, consistent with their high intrinsic resistance to sotorasib. Taken together, this suggested that these two models represent two different classes of resistance: a small number of NCI-H358 cells acquired resistance through an adaptive response, whereas a subpopulation of NCI-H23 cells was intrinsically resistant to sotorasib.

To identify vulnerabilities shared by these two resistant models in an unbiased manner, we screened parental and resistant cells with a curated library of 720 small molecules encompassing major antitumor mechanisms of action and including a series of TEAD inhibitors containing GNE-7883. We compared the mean viability of the parental cell line in standard media versus the resistant model in the media containing sotorasib. Agents targeting TEADs and the PI3K (phosphatidylinositol 3-kinase)/AKT (AKT serine/threonine kinase 1) pathway showed the strongest difference between parental and resistant lines in both cell lines (FDR < 0.002; median decrease of mean viability > 0.15; Fig. 4d), indicating that these resistant cells are more dependent on YAP, TAZ and TEAD. The activation of YAP/TAZ as a mechanism of resistance to sotorasib was further supported by a strong translocation of YAP to the nucleus in sotorasib-resistant NCI-H358 (NCI-H358-R) cells compared with parental control (NCI-H358-P) cells (Extended Data Fig. 5d). Furthermore, changes in chromatin accessibility, as measured by ATAC-seq, revealed that regions opening in sotorasib-resistant cells, relative to parental cells treated acutely with sotorasib, were enriched in the TEAD motif (FDR < 1 × 10^−4^; Fisher’s exact test) and that the genes associated with the regions of increased accessibility were enriched in YAP/TAZ target genes (Fig. 4e). Moreover, gene set enrichment analysis (GSEA)^48^ showed that YAP/TAZ target genes are significantly enriched among the upregulated genes in sotorasib-resistant cells compared with parental cells treated acutely with sotorasib (*P* < 0.001; Fig. 4f). These results support the role of GNE-7883 in overcoming resistance to sotorasib by inhibiting the transcriptional program downstream of YAP, TAZ and TEAD.

**Fig. 5:**
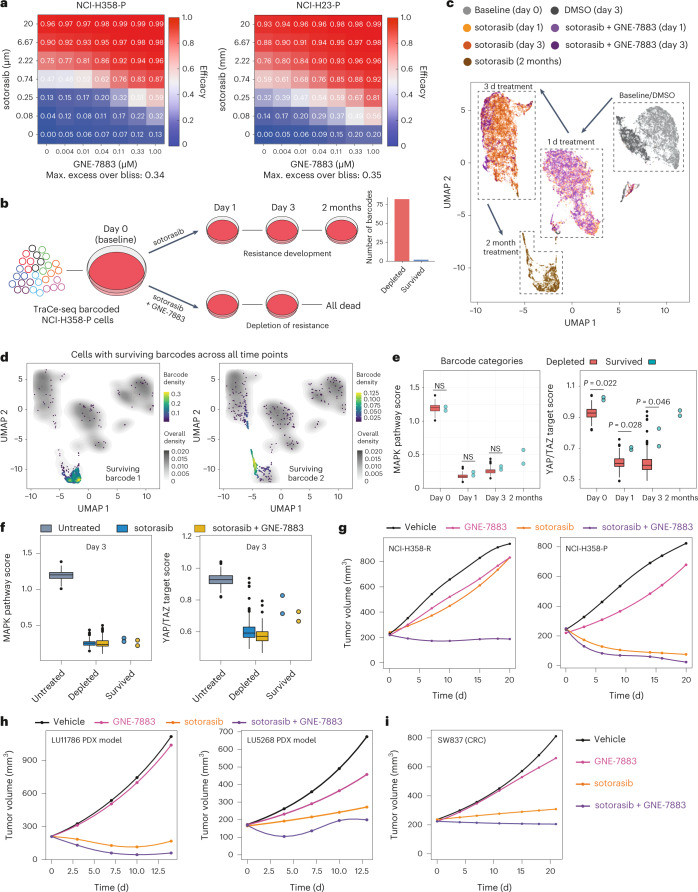
GNE-7883 overcomes sotorasib resistance by suppressing the reactivation of YAP/TAZ target genes. **a**, Heatmaps showing the efficacy (1 for maximum efficacy and 0 for no efficacy) across a dose–response matrix of GNE-7883 and sotorasib combination in NCI-H358 (left) and NCI-H23 (right) cells in 7 d viability assays. **b**, Schematic of the TraCe-seq setup to compare the response of NCI-H358 cells to sotorasib treatment alone versus in combination with GNE-7883. The bar graph shows the number of barcodes that were depleted versus survived after 2 months of sotorasib treatment. **c**, Uniform manifold approximation and projection visualization of all cells collected across all time points and conditions. **d**, Density plot visualization of the distribution of cells carrying either of the surviving barcodes across all time points and treatments. **e**, Plots showing the MAPK pathway (left) and YAP/TAZ target scores (right) for NCI-H358 cells belonging to different barcode categories (*n* = 82 distinct depleted barcodes and *n* = 2 distinct survived barcodes) at baseline (day 0) or under treatment. **f**, Comparison of the MAPK pathway (left) and YAP/TAZ target scores (right) for each NCI-H358 barcode category (*n* = 82 distinct depleted barcodes and *n* = 2 distinct survived barcodes) treated for 3 d with sotorasib alone or in combination with GNE-7883. For **e** and **f**, the boxes (for *n* > 3 only) represent the distribution of scores per barcode for all barcodes in a given category (the counts are aggregated based on the cell barcodes and the box plots comprise the pseudo-bulk values for barcodes in the different categories). The lower and upper hinges correspond to the first and third quartiles (that is, the 25th and 75th percentiles). The lower and upper whiskers extend from the hinge to the smallest or largest value no further than 1.5× the interquantile range from the hinge. The data beyond the ends of the whiskers are outlying points and have been plotted individually. *P* values were calculated by two-sided Wilcoxon rank-sum test. NS, not significant (*P* > 0.05). **g**–**i**, Fitted tumor volumes of the sotorasib-resistant NSCLC NCI-H358-R xenograft model (**g**; left; *n* = 7 mice per group) and sotorasib treatment-naive NSCLC NCI-H358-P xenograft model (**g**; right; *n* = 10 mice per group), NSCLC PDX LU11786 (**h**; left; *n* = 5 mice per group) and LU5268 models (**h**; right; *n* = 5 mice per group) and colorectal SW837 xenograft model (**i**; *n* = 10 mice per group) treated with sotorasib, GNE-7883 or both in combination. Source data

Since GNE-7883 was potent in treating sotorasib-resistant cells, we set out to address whether GNE-7883 could enhance the efficacy of sotorasib in treatment-naive cells. Indeed, GNE-7883 synergized strongly with sotorasib, as measured by Bliss scores^49^, at clinically relevant (submicromolar) concentrations (Fig. 5a).

### GNE-7883 blocks sotorasib resistance in vitro and in vivo

**Extended Data Fig. 6:**
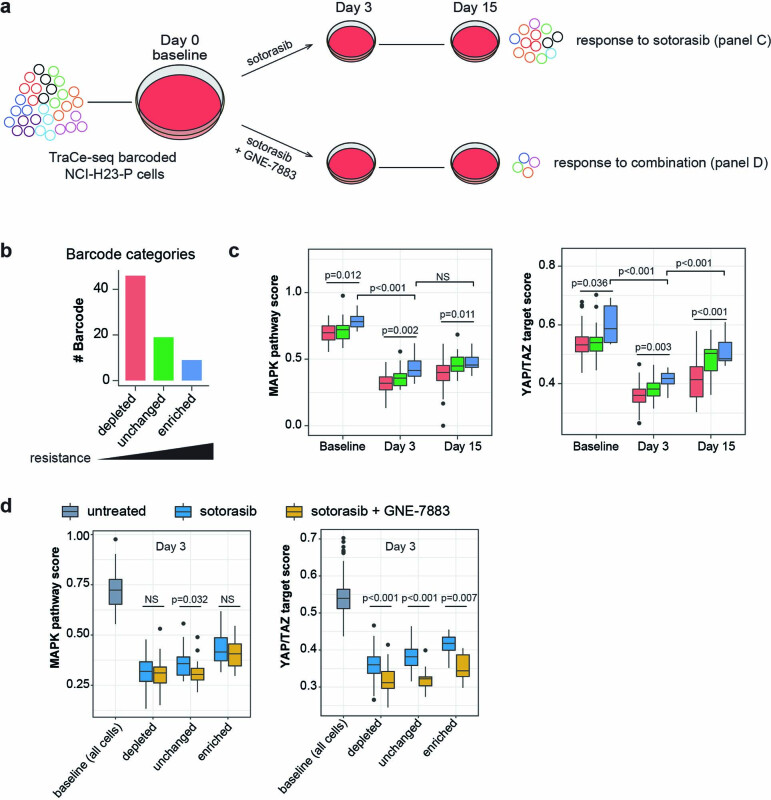
GNE-7883 suppresses sotorasib resistance in NCI-H23 cells. **(a)** Schematic illustrating TraCe-seq setup comparing the response of NCI-H23 cells to sotorasib treatment alone versus in combination with GNE-7883. **(b)** Number of TraCe-seq barcodes belong to each of the categories. **(c)** The MAPK pathway (left) and YAP/TAZ target (right) scores for NCI-H23 cells belonging to different barcode categories (n = 28 distinct depleted barcodes, n = 19 distinct unchanged barcodes, n = 9 distinct enriched barcodes) at baseline (Day 0) or under treatment for 3 or 15 days. Boxes represent the distribution of scores per barcode (average of all cells with the same barcode) for all barcodes in a given category. **(d)** Comparison of the MAPK pathway (left) and YAP/TAZ target (right) scores for each NCI-H23 barcode categories (n = 28 distinct depleted barcodes, n = 19 distinct unchanged barcodes, n = 9 distinct enriched barcodes) treated for 3 days with sotorasib alone or in combined with GNE-7883. For panels (c) and (d), Boxes represent the distribution of scores per barcode for all barcodes in a given category (Counts are aggregated based on the cell barcodes and the box plots comprise the pseudo-bulk values for barcodes in the different categories). Lower and upper hinges correspond to the first and third quartiles (the 25th and 75th percentiles). The lower and upper whiskers extend from the hinge to the smallest or largest value no further than 1.5 × interquantile range (IQR) from the hinge. Data beyond the end of the whiskers are called ‘outlying’ points and are plotted individually. p-values were derived from two-sided Wilcoxon’s rank sum tests between time points, and from Kruskal-Wallis one-way analysis of variance between categories for each time point. NS, not significant (p > 0.05). Source data

To further characterize how GNE-7883 synergizes with sotorasib, we used TraCe-seq^50^ to track the origin, fate and adaptive transcriptional changes in NCI-H23 and NCI-H358 cells. We started with 100 NCI-H358 or NCI-H23 cells, each carrying a unique TraCe-seq barcode, and expanded these initial 100-cell populations in culture to establish TraCe-seq experimental cell populations of NCI-H358 and NCI-H23, respectively (Fig. 5b and Extended Data Fig. 6a). We profiled a fraction of the experimental populations by single-cell RNA sequencing (scRNA-seq) to capture the relative clonal abundance and transcriptional profile of each barcoded clone immediately before drug treatment. In parallel, we treated the remaining cells with sotorasib alone or in combination with GNE-7883 until resistant clones emerged under sotorasib single-agent treatment. We conducted scRNA-seq profiling of the treated cells at intermediate and final time points to capture and compare the transcriptional responses of treatment-sensitive versus treatment-resistant clones (Fig. 5b and Extended Data Fig. 6a). In NCI-H358 cells, adaptation to sotorasib was slow and sparse: only two distinct clones survived and expanded into the drug-resistant population over 2 months (Fig. 5b–d). Consistent with our western blot analysis, the MAPK pathway score^51^ was strongly suppressed by sotorasib across all time points in all clones, with no significant difference between the depleted and survived clones (*P* > 0.1; rank-sum test; Fig. 5e). In stark contrast, cells from the survived clones not only had significantly higher YAP/TAZ target gene expression at baseline (*P* = 0.022; rank-sum test), but also showed substantial reactivation of the YAP/TAZ target genes after their initial downregulation by sotorasib (Fig. 5e). Combination with GNE-7883 effectively wiped out the entire population within 1 week. At the expression level, while GNE-7883 co-treatment did not affect MAPK target gene expression, the combination downregulated YAP/TAZ target genes in cells that could survive sotorasib single-agent treatment (Fig. 5f), indicating that the combination probably acts through YAP/TAZ suppression.

As shown earlier, NCI-H23 represents a treatment refractory model, with brief cytostasis and quick emergence of outgrow under sotorasib treatment. We observed a fourfold increase in cell number over 15 days, probably resulting from differential responses of different subpopulations. We were able to capture such different responses based on the relative clonal abundance changes upon sotorasib single-agent treatment and classified the NCI-H23 clones into three categories: depleted (at least a twofold decrease in relative frequency); unchanged; and enriched (at least a twofold increase in relative frequency), representing increasing levels of sotorasib resistance (Extended Data Fig. 6b). We compared the expression level of MAPK and YAP/TAZ target genes and found that MAPK target genes were suppressed by sotorasib in all three categories and no rebound was observed at day 15 (Extended Data Fig. 6c). In contrast, we observed significant (*P* < 0.001; rank-sum test) reactivation of YAP/TAZ target genes at day 15 across all barcode categories and the reactivation was most prominent in the enriched category (Extended Data Fig. 6c). This suggests that YAP/TAZ activation underlies the tolerance of KRAS G12C inhibition in NCI-H23 cells across all clone categories, and higher YAP/TAZ output is associated with more robust cell growth. The combination with GNE-7883 induced substantial cell death, decreased the cell number by tenfold by day 15 and eventually eliminated all of the remaining cells. At the transcriptional level, GNE-7883 co-treatment did not alter sotorasib’s effects on MAPK target genes, but significantly suppressed YAP/TAZ target genes (*P* < 0.01; rank-sum test; Extended Data Fig. 6d). Collectively, these experiments establish YAP/TAZ activity as a key driver of sotorasib resistance in both NCI-H358 and NCI-H23 models, and demonstrate that the potent pan-TEAD inhibitor GNE-7883 can overcome both adaptive and intrinsic resistance to sotorasib by suppressing the activation of YAP, TAZ and TEAD.

**Extended Data Fig. 7:**
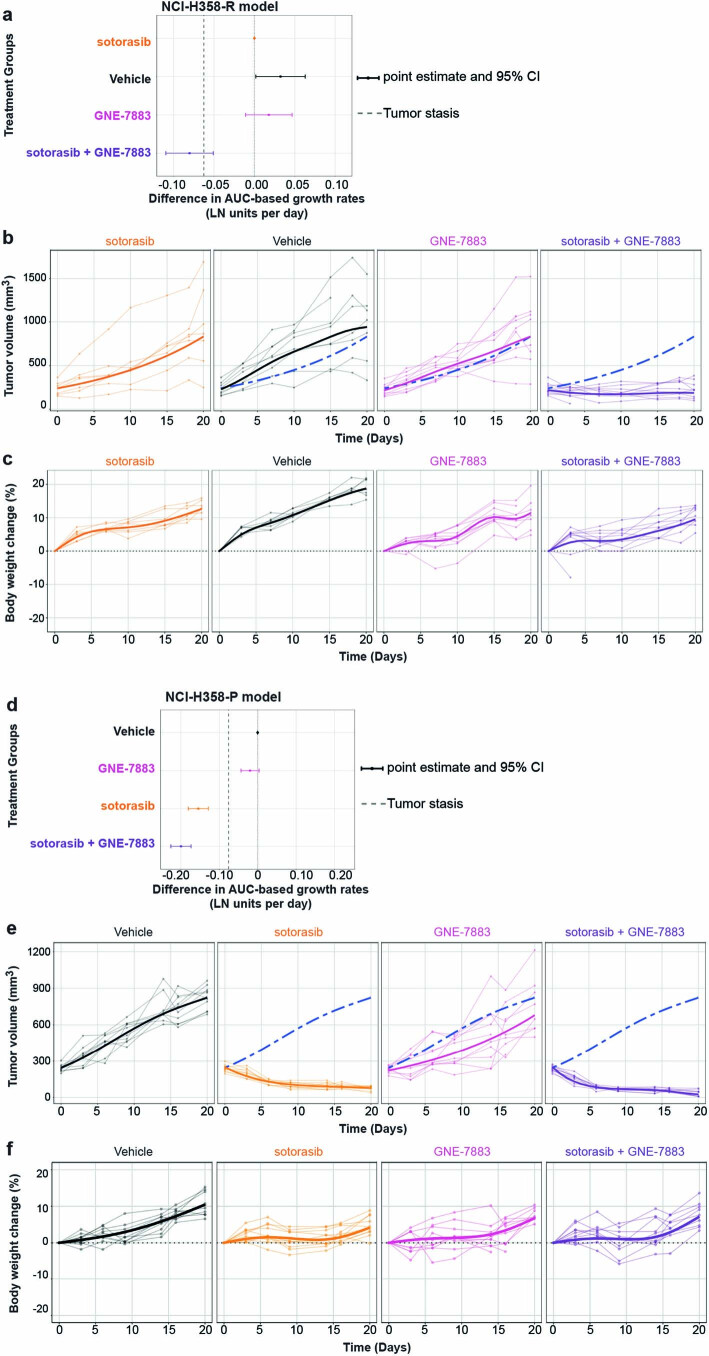
GNE-7883 overcomes intrinsic and acquired sotorasib-resistance in NCI-H358 model *in vivo*. **(a)** Growth contrast plot comparing growth rates (point estimate ± 95% confidence interval) of NCI-H358-R tumors treated with the indicated compounds. n = 8 animals per group. **(b)** Fitted NCI-H358-R tumor volume estimates are shown alongside tumor measurement of individual animals (n = 7 mice per group) over time. Dashed blue lines in the three right plots represent the fitted tumor growth curve under sotorasib treatment. **(c)** Percent changes in body weight of NCI-H358-R tumor-bearing mice (n = 7 mice per group) treated with indicated compounds over time. **(d)** Growth contrast plot comparing growth rates (point estimate ± 95% confidence interval) of NCI-H358-P tumors treated with the indicated compounds. The difference was statistically significant between sotorasib + GNE-7883 combo and sotorasib single agent (Dunnett’s test). **(e)** Fitted NCI-H358-P tumor volume estimates are shown alongside tumor measurement of individual animals (n = 10 mice per group) over time. Dashed blue lines in the three right plots represent the fitted tumor growth curve under vehicle treatment. **(f)** Percent changes in body weight of NCI-H358 xenograft tumor-bearing mice (n = 10 mice per group) treated with indicated compounds over time. Source data

**Extended Data Fig. 8:**
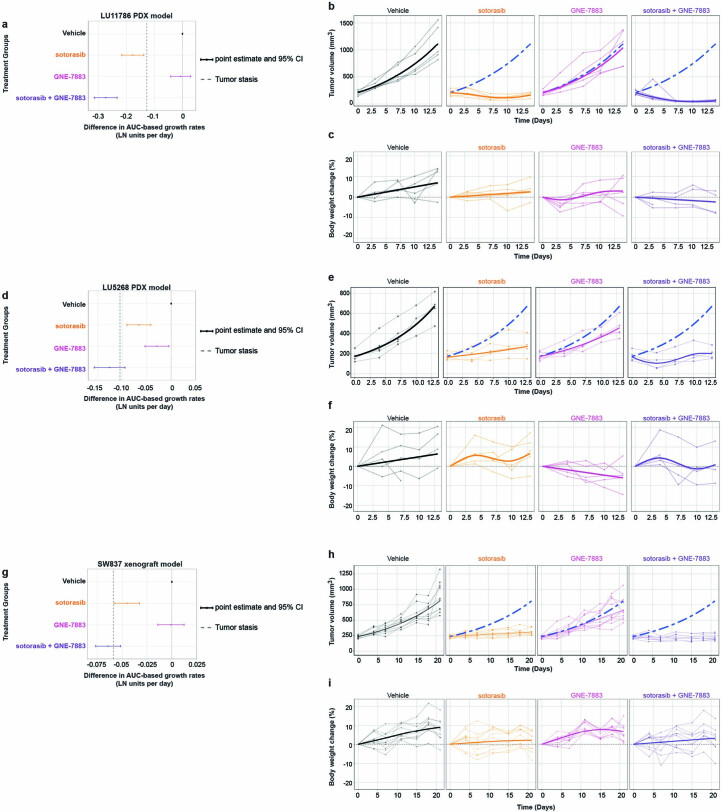
GNE-7883 enhances sotorasib response in PDX and SW837 CRC xenograft models *in vivo*. **(a)** Growth contrast plot comparing growth rates (point estimate ± 95% confidence interval) of PDX LU11786 tumors treated with the indicated compounds. n = 5 mice per group. The difference was statistically significant between sotorasib + GNE-7883 combo and sotorasib single agent (Dunnett’s test). **(b)** Fitted PDX LU11786 tumor volume estimates are shown alongside tumor measurement of individual animals (n = 5 mice per group) over time. Dashed blue lines in the three right plots represent the fitted tumor growth curve under vehicle treatment. **(c)** Percent changes in body weight of PDX LU11786 tumor-bearing mice (n = 5 mice per group) treated with indicated compounds over time. **(d)** Growth contrast plot comparing growth rates (point estimate ± 95% confidence interval) of PDX LU5268 tumors treated with the indicated compounds. The difference was statistically significant between sotorasib + GNE-7883 combo and sotorasib single agent (Dunnett’s test). n = 5 mice per group. **(e)** Fitted PDX LU5268 tumor volume estimates are shown alongside tumor measurement of individual animals (n = 5 mice per group) over time. Dashed blue lines in the three right plots represent the fitted tumor growth curve under vehicle treatment. **(f)** Percent changes in body weight of PDX LU5268 tumor-bearing mice treated with indicated compounds (n = 5 mice per group) over time. **(g)** Growth contrast plot comparing growth rates (point estimate ± 95% confidence interval) of SW837 tumors treated with the indicated compounds. n = 10 mice per group. The difference was statistically significant sotorasib + GNE-7883 combo and sotorasib single agent (Dunnett’s test). **(h)** Fitted SW837 tumor volume estimates are shown alongside tumor measurement of individual animals (n = 10 mice per group) over time. Dashed blue lines in the three right plots represent the fitted tumor growth curve under vehicle treatment. **(i)** Percent changes in body weight of SW837 xenograft tumor-bearing mice treated with indicated compounds (n = 10 mice per group) over time. Source data

To evaluate the efficacy and tolerability of the GNE-7883 and sotorasib combination in vivo, we implanted sotorasib-resistant NCI-H358-R cells into immunocompromised mice and treated established tumors with individual single agents or their combination. While the tumors continued to grow under single-agent treatments, the combination resulted in a significant antitumor effect (Fig. 5g and Extended Data Fig. 7a,b), indicating that the pan-TEAD SMI GNE-7883 can overcome acquired resistance to the KRAS G12C inhibitor in vivo. Next, we asked whether the KRAS G12C inhibitor and GNE-7883 combination could enhance the efficacy of the KRAS G12C inhibitor in treatment-naive models, given that YAP, TAZ and TEAD reactivation underlies adaptation to KRAS G12C inhibitor treatment (Fig. 5d–f). We assessed the combination in the KRAS G12C inhibitor treatment-naive NCI-H358-P xenograft model. Similar to what was observed in the sotorasib-resistant NCI-H358-R model, the GNE-7883 combination significantly (Dunnett’s test based on the area under the curve (AUC) growth rate; Extended Data Fig. 7d) enhanced tumor growth inhibition and led to dramatic tumor regression (Fig. 5g and Extended Data Fig. 7d,e). In both models, treatment was tolerated across all groups (Extended Data Fig. 7c,f). To further strengthen these findings, we evaluated a sotorasib and GNE-7883 combination in patient-derived xenograft (PDX) models LU11786 and LU5268. Similar to what was observed in NCI-H358 xenograft tumors, the combination significantly (Dunnett’s test based on the AUC growth rate; Extended Data Fig. 8a,d) enhanced the efficacy compared with single-agent treatment and was tolerated (Fig. 5h and Extended Data Fig. 8a–f).

Beyond NSCLC, KRAS G12C mutations are found in a variety of solid tumors. Compared with NSCLC, colorectal cancers (CRCs) associated with the KRAS G12C mutation are much less responsive to sotorasib and other KRAS G12C inhibitors in clinical trials^47^. These clinical observations indicate that CRCs appear to be intrinsically more resistant to KRAS G12C inhibition. To assess whether pan-TEAD SMIs could enhance the activity of KRAS G12C inhibitors in this more difficult to treat indication, we turned to colorectal SW837 xenograft models following the same doses as those used for the NCI-H358-R model. Consistent with the heightened intrinsic resistance observed in KRAS G12C CRCs, SW837 xenograft tumors continued to grow under single-agent sotorasib treatment. In contrast, the sotorasib and GNE-7883 combination led to a robust anti-tumor response (Extended Data Fig. 8a,g) and better efficacy compared to sotorasib single agent treatment (Fig. 5i and Extended Data Fig. 8g–i).

## Discussion

In summary, we discovered GNE-7883—part of a pyrazolopyrimidinone-based SMI series—to be a potent and selective compound capable of modulating YAP/TAZ target genes through the inhibition of YAP/TAZ interaction with TEADs. These compounds represent a distinct class of TEAD inhibitors that allosterically block the binding of YAP/TAZ with all four TEAD paralogs through a defined site and mechanism. Mechanistically, GNE-7883 decreases the chromatin accessibility at TEAD motifs and suppresses the transcription of YAP/TAZ target genes. We demonstrate that GNE-7883 is efficacious in cancers harboring genetic alterations in the Hippo pathway and with heightened YAP/TAZ activities, further substantiating the critical role of YAP/TAZ for cancer growth. Beyond its use as a single agent in targeting YAP/TAZ-dependent cancers, we demonstrate that GNE-7883 suppresses the adaptive and intrinsic activation of YAP/TAZ target genes in the context of resistance to the KRAS G12C inhibitor sotorasib, and this combination was found to be highly efficacious and tolerated in a variety of xenograft models.

One limitation of GNE-7883 is its suboptimal pharmacokinetic properties and the requirement to dose subcutaneously in rodents. In addition, despite major efforts across industry and academia to develop YAP/TAZ inhibitors over the past decade, existing preclinical in vivo studies were solely reliant on xenograft cell line models. This pitfall is due to the lack of robust PDX models that are driven by genetic alterations in the Hippo pathway. As an alternative, we leveraged our understanding of the role of YAP, TAZ and TEAD in KRAS G12C inhibitor resistance, and provide proof-of-concept in vivo activity of GNE-7883 and sotorasib combination in KRAS G12C mutant PDX models. These PDX studies provide strong rationale for testing pan-TEAD inhibitors in human clinical trials. We recognize that successful execution of human clinical trials will help to eventually realize the full potential of this class of molecules in precision oncology.

## Methods

### Ethics statement

Our research complies with all of the relevant ethical regulations. Animals were maintained in accordance with the Guide for the Care and Use of Laboratory Animals (National Research Council, 2011). Genentech is an Association for Assessment and Accreditation of Laboratory Animal Care-accredited facility and all animal activities in this research study were conducted under protocols approved by the Genentech Institutional Animal Care and Use Committee. The maximum tumor size permitted by the ethics committee/institutional review board is 2 cm^3^. This maximum tumor size/burden was never exceeded in the studies. Source data are provided for all of the in vivo experiments.

### Cell lines, antibodies and other reagents

All of the cell lines used in this study were obtained from the American Type Culture Collection or the Genentech cell bank. They were maintained in RPMI 1640 supplemented with 10% fetal bovine serum (Sigma–Aldrich) and 2 mM l-glutamine (Gibco) in a humidified incubator maintained at 37 °C with 5% CO_2_. Cell line authentication was conducted as previously described^29^, specifically for short tandem repeat profiling using the Promega PowerPlex 16 System. This was performed when receiving new cell lines and the results were compared with external short tandem repeat profiles of cell lines (when available) to determine cell line ancestry. Cell line authentication was routinely conducted via single-nucleotide polymorphism-based genotyping using Fluidigm multiplexed assays at the Genentech cell line core facility. Cells were assessed with a Vi-CELL Cell Viability Analyzer (Beckman Coulter). A viability of at least 90% was required for experiments and screening.

The antibodies used in this study included pan-TEAD (13295; CST; 1:500), YAP (14074; CST; 1:500), TAZ (70148; CST; 1:500), YAP/TAZ (8418; CST; 1:500), MAX (sc-765 and sc-8011; Santa Cruz; 1:500), α-tubulin (3873; CST; 1:10,000), β-actin (3700; CST; 1:10,000), cleaved PARP (9541; CST; 1:3,000), p21 (2947; CST; 1:500), anti-rabbit and anti-mouse HRP linked (7074 and 7076; CST; 1:20,000), IRDye anti-rabbit and anti-mouse (68070 and 32211; LI-COR; 1:20,000), KRAS Rb pAb (12063-1-AP; Proteinech; 1:1,000), Phospho-S6 Ribosomal Protein (Ser235/236) (2211; CST; 1:1,000), S6 Ribosomal Protein (5G10) Rabbit mAb (2217; CST; 1:1,000), Phospho-MEK1/2 (Ser217/221) (41G9) Rabbit mAb (9154; CST; 1:1,000), Phospho-p44/42 MAPK (Erk1/2) (Thr202/Tyr204) (D13.14.4E) XP Rabbit mAb (4370; CST; 1:1,000), p44/42 MAPK (Erk1/2) (3A7) Mouse mAb (9107; CST; 1:1,000), Phospho-p90RSK (Ser380) (D5D8) Rabbit mAb (12032; CST; 1:1,000), Purified Mouse Anti-Rsk Clone 78/RSK (610226; BD Biosciences; 1:1,000), Vimentin (5G3F10) Mouse mAb (3390s; CST; 1:1,000), Mouse Anti-MEK1 Clone 25/MEK1 (610122; BD Biosciences; 1:1,000), Phospho-Akt (Ser473) (D9E) XP Rabbit mAb (4060; CST; 1:1,000) and Akt (pan) (40D4) Mouse mAb (2920; CST; 1:1,000).

Experimental compounds and sotorasib were synthesized by Genentech. Detailed descriptions of the compound structures and chemistry information for experimental compounds are provided as Supplementary Data.

### Cell viability and colony formation assays

For pan-TEAD experimental compound treatment, cells were seeded at 1,000 cells per well in U-bottom ultra-low-adhesion 96-well plates and were treated after 12–24 h with experimental compounds. For sotorasib and sotorasib plus GNE-7883 treatments, cells were plated at a concentration of 20,000 cells per ml in 96- or 384-well plates. At 24 h post-plating, cells were treated with a nine-point titration (1:3) of the desired chemical compounds using the HP D300 drug dispenser. Cell growth was assessed using CellTiter-Glo Luminescent Cell Viability Assays (Promega) and the luminescence was read with a 2104 EnVision Multilabel Plate Reader (PerkinElmer). All cell viability data were collected and calculated for at least five replicates per time point per condition. IC_50_ values for the inhibitors were determined by fitting the nonlinear regression curves generated by GraphPad Prism.

For combination synergy studies, cells were plated in 384-well plates (Corning) and treated with varying concentrations of compounds, either alone or in combination for 7 days. Cell viability was determined using CellTiter-Glo Luminescent Cell Viability Assay (G7573; Promega). Synergistic effects were determined using the Bliss independence analysis methods^49^.

To assess colony formation, cells were seeded at 1.5 × 10^3^ cells per well in 40 μl 0.29% soft agar on a base layer of 40 μl 0.6% soft agar and treated with 40 μl media containing 3× concentrated experimental compound. At day 7, 40 μl media containing 4× concentrated experimental compound was added to each well. Colonies were imaged and counted on days 7, 10 and 14 using a GelCount colony counter (Oxford Optronix) per the manufacturer’s instructions.

### TEAD reporter assay

#### Stable reporter line generation and maintenance

MDA-MB-231 cells were transfected with a reporter plasmid containing a nano-luciferase reporter element under the control of the Hippo pathway response element TEAD. As a counter-screen, the plasmid also contained firefly luciferase under the control of the PGK (phosphoglycerate kinase) promoter, which is unrelated to the Hippo pathway. Following transfection and dilution cloning, individual clones were selected and characterized. Clones were grown and maintained in RPMI 1640, 10% fetal bovine serum, 2 mM l-glutamine and 50 µg ml^−1^ Zeocin (Invitrogen).

#### Reporter assay with test compounds

Cells were plated (day 1) in 384-well tissue culture-treated assay plates and incubated overnight. Two cell plates were prepared for each compound plate. The following day (day 2), cells were treated with compounds and incubated overnight. On day three, cell plates were incubated with either Nano-Glo luciferase reagent (N1110; Promega), for on-target determination of pathway inhibition, or Firefly luciferase reagent (E8110; Promega), for the determination of off-target activity of compounds. Luminesence measurements were taken on a 2104 EnVision Multilabel Plate Reader (PerkinElmer). Duplicate ten-point dose–response curves were generated for each test compound. The potencies of compounds as Hippo pathway inhibitors were determined by IC_50_ values generated using a nonlinear four-parameter curve fit.

### Immunoprecipitation and immunoblotting

Immunoprecipitation and immunoblotting were conducted as previously described^29^. Specifically, cells were lysed in RIPA buffer (Thermo Fisher Scientific) containing protease inhibitor (Roche) and phosphatase inhibitor (Roche). Lysates were prepared by taking supernatants from centrifugation at 12,000*g* and 4 °C for 15 min. Equivalent amounts of proteins were loaded and separated by sodium dodecyl sulfate polyacrylamide gel electrophoresis, followed by transfer to membranes. For the endogenous co-immunoprecipitation experiments, 1 × 10^7^ cells were lysed using RIPA buffer (Thermo Fisher Scientific) and immunoprecipitation was performed with the indicated antibody overnight at 4 °C. After washing with RIPA buffer (Thermo Fisher Scientific), co-immunoprecipitated endogenous proteins were detected by immunoblotting.

### Subcellular fractionation

Cells grown on 10 cm dishes were harvested after washing with cold phosphate-buffered saline (PBS). Cell pellets were then incubated with 400 μl buffer A (10 mM HEPES (pH 7.9), 10 mM KCl, 1 mM EDTA, 0.1 mM EGTA, 0.2% NP-40 and 10% glycerol) on ice for 20 min. After centrifugation for 30 s at 1,400*g*, cytoplasmic fractions were collected by retaining the supernatant. For the preparation of nuclear fractions, pellets were washed in 400 μl buffer A, then incubated with 200 μl RIPA buffer for 30 min at 4 °C. After centrifugation for 12 min at 16,000*g*, nuclear fractions were collected by supernatant retainment.

### RNA analyses

For treatment, cells were seeded at 1–2 × 10^5^ cells per well on tissue culture-treated 6-well plates and treated after 24 h with experimental compound. Cell lines were lysed on the plates for RNA isolation using the Qiagen RNeasy Plus Mini kit (catalog number 74034) and the RNA concentration was determined using a NanoDrop 8000 (Thermo Fisher Scientific).

For analysis by RNA Fluidigm, 100 ng RNA was subjected to a complementary DNA (cDNA) synthesis reaction using the Applied Biosystems High-Capacity cDNA Reverse Transcription Kit (4368814; Thermo Fisher Scientific), per the manufacturer’s protocol. A preamplification reaction was then performed with Taqman PreAmp Master Mix (Thermo Fisher Scientific). After amplification, samples were diluted 1:4 with Tris-EDTA, and quantitative PCR (qPCR) was conducted on Fluidigm 96.96 Dynamic Arrays using the BioMark HD system according to the manufacturer’s instructions. Fluidigm data were analyzed with RealTime StatMiner for qPCR in the Spotfire program.

RNA-seq was performed as previously described^29^. Specifically, RNA integrity was confirmed using a Bioanalyzer 2100 (Agilent Technologies). About 500 ng RNA was used for library synthesis with the TrueSeq RNA Sample Preparation Kit v2 (Illumina). The size of the libraries was confirmed using a 2200 TapeStation and High Sensitivity D1K ScreenTape (Agilent Technologies) and the concentration was determined via a qPCR-based method using the Library Quantification Kit (KAPA). The libraries were multiplexed and then sequenced on an Illumina HiSeq 2500 (Illumina) to generate 30 million single-end 50-base pair (bp) reads. For RNA-seq data analyses, RNA-seq reads were first aligned to ribosomal RNA sequences to remove ribosomal reads. The remaining reads were aligned to the human reference genome (GRCh38) using GSNAP version 2013-10-10, allowing a maximum of two mismatches per 75-base sequence (parameters: -M 2 -n 10 -B 2 -i 1 -N 1 -w 200000 -E 1 —pairmax-rna = 200000—clip-overlap)^52^. Transcript annotation was based on the Ensembl genes database (release 77). To quantify gene expression levels, the number of reads mapped to the exons of each RefSeq gene was calculated. Differential gene expression was performed with DESeq2. A prefilter was applied so that only genes with at least a median number of reads per kilobase per million mapped reads of ten in one were analyzed. *P* values for other genes were simply set to 1 and log[fold change] values were set to 0 for visualization purposes, but such genes were not included in the multiple testing correction. *Q* values were obtained by correcting *P* values for multiple hypotheses using the Benjamini–Hochberg procedure. Genes were considered if they had a *Q* value of <0.05 and were protein coding. Counts were transformed to log_2_[counts per million], quantile normalized and precision weighted with the voom function of the limma package.

### ATAC-seq library preparation

ATAC-seq was performed essentially as described previously^53^. Briefly, cryo-preserved cells were thawed, washed with 1× PBS and counted. About 100,00 cells were then lysed for 5 min on ice in 100 µl lysis buffer (10 mM Tris (pH 7.5), 10 mM NaCl, 3 mM MgCl_2_, 0.1% NP-40, 0.1% Tween-20 and 0.01% digitonin). 1 ml wash buffer (10 mM Tris (pH 7.5), 10 mM NaCl, 3 mM MgCl_2_ and 0.1% Tween-20) was then added and the pellets were spun down at 500*g* for 10 min at 4 °C. The supernatant was discarded and the pellet was resuspended in 50 µl tagmentation buffer (25 µl 2× TD buffer, 16.5 µl PBS, 0.5 µl 10% Tween-20, 0.5 µl 1% digitonin, 2.5 µl Tn5 transposase and 5 µl H_2_O). The tubes were incubated at 37 °C for 30 min, followed by DNA isolation using the Qiagen MinElute Cleanup Kit (catalog number 28206). The DNA was then amplified using NEBNext 2× PCR Master mix (catalog number M0541L) and analyzed by TapeStation (Aiglent) before submission for sequencing.

### ATAC-seq analysis

ATAC-seq reads were analyzed using the ENCODE ATAC-seq pipeline (version 1). Briefly, reads were trimmed of adapters using cutadapt (version 1.9.1) and mapped to hg38 using Bowtie 2 (version 2.2.6). Bam files were converted to tagAlign format, which was then adjusted for Tn5 by shifting +4 bp for positive strands and −5 bp for negative strands. TagAlign files were used to call peaks using MACS2 (version 2.1.0), and those peaks with *P* < 1 × 10^−6^ were retained for differential analysis with DiffBind (version 3.0.13) for Fig. 1 or the bamCount function of the R package bamsignals (version 1.24.0) for Fig. 3. For subsequent comparative analyses, only peaks called in at least two samples were retained. Differential accessibility analysis was performed using either the R package DiffBind (version 3.0.13) for Fig. 1 or DESeq2 (version 1.32.0) for Fig. 3. Differential peaks were defined by an absolute fold change of >2 and called peaks by MACS2 in at least two samples with gained accessibility (the Benjamini–Hochberg procedure was used for multiple hypothesis correction with a cut-off of FDR < 0.01 for Fig. 1 and FDR < 0.1 for Fig. 3). The genomic distribution of differential peaks was annotated with ChIPpeakAnno (version 3.24.1). Bigwigs corresponding to a fold change against the background control were generated by MACS2 and used to generate heatmaps with the heatmap function of deepTools (version 3.5.0). Motif enrichment analysis was performed using either HOMER (version 4.10) with all peaks as the background for Fig. 1 or the AME (version 5.4.1) tool of the MEME suite using the nondifferential ATAC-seq peaks as background and the HOCOMOCOv11_core_HUMAN motif database. The peaks were assigned using Poly-Enrich with distal enhancer–gene target links (>5 kilobases from the transcription start site) plus 5-kilobase locus definitions. Gene set enrichment was searched against Gene Ontology, MsigDB Oncogenic and Hallmark.

### Statistical analysis

The RNA-seq studies are analyzed as stated above. Statistical analysis for the other in vitro studies was performed using two-tailed, unpaired Student’s *t-*tests or as indicated in the figure legends. All of the in vitro experiments were repeated at least three times. A *P* value of <0.05 was considered statistically significant.

### TEAD lipid pocket TR-FRET assay

His-tagged TEAD proteins (YAP-binding domain) were preincubated with compounds for 30 min at room temperature. Biotinylated lipid pocket probes were then added to the TEAD and compound mixture and incubated for 60 min at room temperature. Next, a europium-labeled anti-His antibody (PerkinElmer) and XL665-labeled streptavidin (Cisbio) were added to the TEAD, compound and probe mixture and incubated for an additional 30 min. TR-FRET values were then measured using a 2104 EnVision Multilabel Plate Reader (PerkinElmer). The potency of compounds was determined by generating an IC_50_ value using a nonlinear four-parameter curve fit.

### TEAD–YAP/TAZ TR-FRET assay

Purified His-tagged TEAD proteins (YAP-binding domain) were preincubated with europium-labeled anti-His antibody tracer (PerkinElmer). The TEAD–europium protein complex was then incubated with small molecules for 30 min. In parallel, biotinylated YAP peptide (amino acids 50–100) was preincubated with streptavidin-XL665 acceptor (Cisbio). The preincubated YAP peptide was then added to the compound and TEAD mix. The TEAD–YAP–inhibitor mixture was then incubated for 60 min at room temperature in a polystyrene plate. At the end of the incubation, the plate was read on a plate reader using TR-FRET mode with wavelengths of 665 and 615 nm. The potencies of compounds were determined by IC_50_ or half-maximal effective concentration value generated using a nonlinear four-parameter curve fit. The extent to which representative examples of the disclosed compounds were able to inhibit the interaction between TEAD1, TEAD2, TEAD3 or TEAD4 and YAP or TAZ was measured by HTRF to generate half-maximal effective concentration data.

### TEAD site 2- and 3-specific biochemical assays and confirmation of site 2 perturbation

To characterize which YAP-binding sites are perturbed by small molecules, TEAD site-specific PPI assays were developed using a previously identified^38^ biotinylated peptide probe based on mouse vestigial-like protein 1 amino acids 20–51 (mVgll1; refs. ^20–51^) corresponding to YAP/TEAD site 2. Additionally, the previously described^39^ site 3-specific peptide 17 was adapted for use as a site 3-specific HTRF probe. In 384-well solid white assay plates (PerkinElmer), purified His-tagged TEAD proteins were preincubated with test compounds for 30 min in an assay buffer containing 50 mM HEPES (pH 7.2), 100 mM NaCl, 0.01% Tween-20, 0.5 mg ml^−1^ bovine gamma globulin and 1 mM dithiothreitol. Following 30 min of TEAD plus compound incubation, either the biotinylated mVgll1 (refs. ^20–51^) site 2 probe or the biotinylated peptide 17 site 3 probe was added and incubated for 60 min. Site 2 and site 3 assays were carried out in separate assay plates. After 60 min, HTRF reagents, including a europium-labeled anti-His antibody TR-FRET donor (PerkinElmer) and streptavidin-coated XL665 TR-FRET acceptor (Cisbio), were added to all wells. Reactions were incubated for an additional 30 min. All incubations were carried out at room temperature. Assay plates were then read on a 2104 EnVision Multilabel Plate Reader (PerkinElmer) using HTRF 665/615 mode. Increased TR-FRET values indicated binding of the mVgll1 (refs. ^20–51^) peptide or peptide 17 to TEAD, whereas decreased values indicated displacement by SMIs.

### NMR spectroscopy

Three synthetic peptides, S2 (^59^GDSETDLEALF_(F2)_NAVMNPKTANVP^81^), S3 (^80^VPQTVPMRLRKLPDSF_(F2)_FKPPE^100^) and pepYAP (^60^DSETDLEALFNAVMNPKTANV PQTVPMRLRKLPDSFFKPPE^100^), encompassing residues 59–81, 80–100 and 60–100 of YAP, respectively, were purchased from ABclonal. Residues Phe69 and Phe95 in the YAP protein were replaced with 3,5-difluoro-phenylalanine (F_(F2)_) in the S2 and S3 peptides. Both S2 and S3 had acetylated amino (N) and carboxy (C) termini. The samples for the NMR studies contained 20 μM peptide S2, 20 μM peptide S3 and 10 μM TEAD2 in PBS (100% D_2_O) at pH 7.4. For the displacement experiments, compound 2, GNE-7883 or pepYAP were added to a final concentration of 55 μM to samples containing S2, S3 and TEAD2. NMR experiments were carried out on a Bruker Avance III spectrometer equipped with a QCI-F CryoProbe. 1D ^19^F spectra were collected with 1,024 scans, an acquisition time of 94.4 ms and ^1^H decoupling. All NMR spectra were acquired at 298 K and referenced internally to trifluoroacetic acid at −76.5 ppm. NMR data were processed and analyzed using the TopSpin package.

### Protein expression and purification

A TEAD1 (S260–D426) construct harboring an N-terminal GST tag with a thrombin cleavage site and a C-terminal 6× His-tag was expressed in insect cells (SF9) by baculoviral expression. Cells were harvested 48 h after infection and lysed by dounce homogenization in lysis buffer (50 mM Tris (pH 8.0), 300 mM NaCl, 5% glycerol, 1 mM TCEP and 1× Roche EDTA-free protease inhibitor cocktail). Insoluble material was removed by ultracentrifugation and cleared lysates were applied to nickel-NTA resin on a gravity column that had been pre-equilibrated in buffer A (lysis buffer containing 30 mM imidazole). Resin was washed with 20 column volumes. Buffer A and sample were eluted with buffer A containing 300 mM imidazole and 2.5 mM CaCl_2_. Thrombin was added to the sample and the sample was dialyzed against buffer A containing 2.5 mM CaCl_2_ overnight. Sample was again applied to a nickel-NTA column that had been pre-equilibrated with buffer A and the flow through was collected. The cleaved sample was then treated with 2.5% hydroxylamine (Sigma–Aldrich) at pH 7.0 for 1 h at room temperature before size exclusion chromatography on a Superdex 200 16/60 column that had been pre-equilibrated in buffer B (20 mM Tris (pH 7.5), 100 mM NaCl, 5% glycerol and 5 mM dithiothreitol).

N-terminally 6× His-tagged TEAD2 (A217–D447), TEAD3 (Q216–D435) and TEAD4 (Q215–E434) with TEV protease cleavage sites were expressed in *Escherichia coli* BL21 (DE3) cells by standard autoinduction and purified as described^30^ with the exception that the proteins were treated with 2.5% hydroxylamine (Sigma–Aldrich) at pH 7.0 for 1 h at room temperature before size exclusion chromatography. For crystallography of TEAD2, the final protein sample was concentrated to 5 mg ml^−1^.

### Crystallization

Crystallization was conducted as previously described^31^. Specifically, TEAD2 crystals of the base-centered monoclinic space group *C*121 were grown at 19 °C by hanging drop vapor diffusion using a drop ratio of 1:1 protein:reservoir solution and streak seeding. Reservoir solution contained 200 mM potassium/sodium tartrate (pH 6.5) and 20% PEG 3350. To obtain co-crystal structures, crystals were soaked for 5 d in 2 μl reservoir solution containing a final concentration of 2 mM compound and 35% PEG 3350. Crystals were then flash frozen directly in liquid nitrogen.

### Data collection and structure determination

X-ray diffraction data for compound 1 and GNE-7883 were collected at beamline 5.0.2 at the Advanced Light Source. Data for compound 2 were collected at beamline 12-2 at the Stanford Synchrotron Radiation Lightsource. Data were processed using autoPROC and elliptically truncated using STARANISO^54–58^. All structures were solved as previously described^31^ using molecular replacement in Phaser^59^. There were two molecules in the asymmetric unit. Structures were rebuilt in Coot^60^ and subjected to iterative rounds of refinement and rebuilding using Phenix^61^ and Coot. Data processing and refinement statistics are summarized in Supplementary Table 2.

### Pharmacokinetic analysis

Pharmacokinetic properties of GNE-7883 were determined in C57BL-6 mice. All mice were female and 5–6 weeks old at the time of the study. GNE-7883 was suspended in sunflower oil (Spectrum Chemical), agitated by water bath sonication and vortexed to generate a homogenous formulation. The mice were administered GNE-7883 at 250 mg kg^−1^ in sunflower oil by subcutaneous injection once daily. Food and water were available ad libitum to all mice. Serial blood samples (15 μl) were collected by tail nick at 0.25, 0.5, 1, 2, 4, 8 or 24 h after administration. Blood samples were diluted with 60 μl water containing 1.7 mg ml^−1^ EDTA and kept at 80 °C until analysis. Plasma concentrations of GNE-7883 were determined by liquid chromatography–tandem mass spectrometry assay.

### Mouse xenograft

Female C.B-17 SCID (inbred) mice were obtained from Charles River Laboratories at Hollister. All of the mice used in the study were female and 7–10 weeks of age at the start of the study. The mice were fed ad libitum with an autoclaved rodent diet (LabDiet 5010). Mice were housed in individually ventilated cages within animal rooms maintained on a 14 h/10 h light/dark cycle. Animal rooms were temperature and humidity controlled, between 20.0 and 26.1 °C and 30 and 70%, respectively, with 10–15 room air exchanges per hour.

For the xenograft studies, NCI-H226, MSTO-211H, NCI-H358 and SW837 cells were cultured in vitro in RPMI 1640 media plus 1% l-glutamine with 10% fetal bovine serum, harvested in log-phase growth and resuspended in Hank’s Balanced Salt Solution containing Matrigel (BD Biosciences) at a 1:1 ratio by volume for in vivo inoculation. C.B-17 SCID mice were subcutaneously inoculated with 10 × 10^6^ NCI-H226 cells in the right flank. Mice were dosed with GNE-7883 (250 mg kg^−1^; 4 d on and 2 d off) in sunflower oil by subcutaneous injection. For the MSTO-211H xenograft study, C.B-17 SCID.bg mice were subcutaneously inoculated with 10 × 10^6^ MSTO-211H cells in the right flank and dosed with GNE-7883 (250 mg kg^−1^; 2 d on and 1 d off) by subcutaneous injection. For the SW837 xenograft study, NSG mice were subcutaneously inoculated with 10 × 10^6^ SW837 cells in the right flank and dosed with GNE-7883 (250 mg kg^−1^; 2 d on and 1 d off) by subcutaneous injection and/or sotorasib (25 mg kg^−1^ daily), in 70% polyethylene glycol 400 (PEG 400) and 1.5% dextrose, once daily by oral gavage. For the NCI-H358-R xenograft study, NCI-H358-R sotorasib-resistant cell lines were derived in vitro from NCI-H358 cells then established for in vivo growth and propagated through subcutaneous tumor transplantation from donor to recipient C.B-17 SCID mice. Upon dosing, mice were given sotorasib (25 mg kg^−1^ daily), formulated in 70% PEG 400 and 1.5% dextrose, once daily by oral gavage and/or G 7883 (250 mg kg^−1^; 2 d on and 1 d off), in 100% sunflower oil, by subcutaneous injection. In both studies, tumors were allowed to grow to a volume in an initial range before mice were randomized to treatment groups at the start of dosing to create closely matched baseline average tumor sizes across regimens. For the PDX studies, mice were implanted with a 2 mm × 2 mm chunk of LU11786 or LU5268 NSCLC tumor in the right front flank and dosed with GNE-7883 (250 mg kg^−1^; 2 d on and 1 d off) by subcutaneous injection and/or sotorasib (50 mg kg^−1^ daily), in 70% PEG 400 and 1.5% dextrose, once daily by oral gavage. All of the PDX studies were conducted at Crown Bioscience in accordance with their standard operating procedures.

In all of the studies, tumors were allowed to grow to a volume in an initial range before mice were randomized to treatment groups at the start of dosing, to create closely matched baseline average tumor sizes across regimens. Mice bearing tumors were evenly distributed into study groups based on the mean tumor volume of the whole cohort so that the standard deviation was equal across all groups. Tumor sizes and mouse body weights were recorded twice weekly over the course of the study. In house, tumor volumes were measured in two perpendicular dimensions (length and width) using Ultra Cal IV calipers (model 54-10-111; Fred V. Fowler). Tumor volumes were then calculated as: tumor size (mm^3^) = (longer measurement × shorter measurement^2^) × 0.5. Body weights were measured using an Adventurer Pro AV812 scale (Ohaus Corporation). Percentage animal weight changes were calculated as: body weight change (%) = [(current body weight/initial body weight) − 1) × 100].

The efficacy studies used five to ten mice per group. The sample sizes were chosen based on historical studies; our models had been run numerous times previously and we have a full understanding of their performance. In addition, we had previously performed dose escalation studies of the compounds used in this manuscript and we have a good understanding of their effects. The PDX studies were blinded. The other xenograft studies were not blinded because it is prohibitive cost wise. Multiple people were involved in running these studies and everyone worked with integrity and honesty. In addition, these models and molecules were tested multiple times by various researchers at Genentech.

Analyses and comparisons of tumor growth were performed using a package of customized functions in R (version 3.6.2; R Foundation for Statistical Computing), which integrates software from open-source packages as described by Forrest et al.^62^. The term growth contrast represents the difference in AUC-based growth rates (endpoint gain integrated in time) between the treatment and reference group^62^. The more negative the growth contrast value, the greater the antitumor effect. The 95% confidence intervals were based on the fitted model and variability measures of the data.

### Compound library screen and analysis

A panel of 720 small-molecule compounds, including targeted agents, chemotherapeutics and tool compounds, was used to treat NCI-H358/NCI-H23 parental cells in standard media and their corresponding resistant model in the media containing 900 nM sotorasib. Compounds were obtained from in-house synthesis or purchased from commercial vendors. Cells were maintained in RPMI 1640, 5% fetal bovine serum and 2 mM glutamine in a humidified incubator maintained at 37 °C with 5% CO_2_. Cells were assessed with a Vi-CELL Cell Viability Analyzer (Beckman Coulter) and a viability of at least 90% was required for screening. A Multidrop Combi Reagent Dispenser (Thermo Fisher Scientific) was used to plate cells into Falcon 384-well, black, clear-bottom plates (353962; Corning) using seeding densities previously determined to achieve approximately 70–80% confluence at the final time point of the assay. On the following day, cells were treated with a nine-point dose titration of the chemical library using a Bravo Automated Liquid Handling Platform (Agilent). After 5 d, 25 ml CellTiter-Glo reagent was added using a MultiFlo Microplate Dispenser (BioTek). Cell lysis was induced by mixing for 30 min on an orbital shaker; plates were then incubated at room temperature for 10 min to stabilize the luminescence signal. The luminescence was read using a 2104 EnVision Multilabel Plate Reader (PerkinElmer). The data were processed using Genedata Screener version 14 (Genedata), with a four-parameter Hill equation using compound dose–response data normalized to the median of 42 vehicle-treated wells on each plate. A robust fit strategy was also employed by Genedata Screener, which was based on Tukey’s biweight and was resistant to outlier data. The reported absolute IC_50_ was the dose at which cross-run estimated inhibition was 50% relative to dimethyl sulfoxide control wells. In addition to the absolute IC_50_, the mean fitted viability across the nine tested doses (that is, the area under the viability curve) was also computed. Compounds were ranked by their differences in mean viability between the resistant lines and parental lines, and target enrichment analysis was performed using the same approach as GSEA^48^.

### TraCe-seq experimental procedure

TraCe-seq experiments were conducted following a similar method to that described previously^50^. Briefly, 10 million NCI-H358 and NCI-H23 cells were infected with a TraCe-seq library^50^ with 100 K barcode complexity at a multiplicity of infection of 0.1. Cells were selected with puromycin and sorted for the top 50% of green fluorescent protein (GFP)-expressing cells by fluorescence-activated cell sorting to establish the TraCe-seq parental barcoded pool. The TraCe-seq parental barcoded pool was passaged once in cultured, trypsinized and dissociated to single cell suspensions, and 100 cells were seeded into a single well of a 96-well tissue culture plate to establish a TraCe-seq population with ~100 clones carrying unique barcodes. These 100 cells were subsequently expanded for 14–15 doublings over 20–25 days to establish the experimental population. The experimental population was then seeded in 6-well tissue culture plates (60,000 cells per well for NCI-H358 and 30,000 cells per well for NCI-H23) and left to attach overnight. The next day, two wells of NCI-H358 cells and three wells of NCI-H23 cells were trypsinized, dissociated into single cells and subject to scRNA-seq. The remaining cells were treated with sotorasib (800 nM for NCI-H358 and 1,500 nM for NCI-H23) alone or in combination with either 500 nM GNE-7883 or dimethyl sulfoxide control. Treated cells were harvested for scRNA-seq at the time points indicated in Fig. 5b (NCI-H358) and Extended Data Fig. 6a (NCI-H23).

### Single-cell RNA-seq

Single-cell RNA-seq was conducted as previously described^50^. Specifically, cultured cells were trypsinized into single-cell suspensions and processed using the Chromium Single Cell 3′ Gene Expression Library and Gel Bead Kit v3.1 following the manufacturer’s instructions (10x Genomics). Cells were counted and checked for viability using a Vi-CELL XR cell counter (Beckman Coulter), then injected into microfluidic chips to form gel beads in emulsion in the 10x Chromium instrument. Reverse transcription was performed on the gel beads in emulsion and the products were purified and amplified. Expression libraries were made from the cDNA, profiled using the Bioanalyzer High Sensitivity DNA kit (Agilent Technologies) and quantified with the KAPA Library Quantification Kit (Kapa Biosystems). Illumina HiSeq 2500 and HiSeq 4000 instruments were used to sequence the libraries.

### TraCe-seq analysis

Single-cell RNA-seq FASTQ files were processed using kallisto (version 0.46.2) and bustools (version 0.40.0) workflows^63^, utilizing a custom index created from the human reference transcriptome (GRCh38; Ensembl 90) including intronic sequences with 30-bp flanking regions and custom transgene GFP sequences fused to one of 100,000 30-bp GC-optimized barcodes. Three gene count matrices (all, spliced and unspliced counts) were generated, containing numbers of unique molecular identifiers for both annotated genes and GFP barcodes. The latter were separated from the former and used to assign GFP barcodes to cells, with cells expressing multiple barcodes being assigned a single one if the top barcode had at least a threefold higher number of counts than other barcodes. For annotated gene counts, all counts were used for all subsequent steps.

Downstream analysis of the results was performed in the R environment (R version 4.1 and Bioconductor version 3.13) following the OSCA book recommendations^64^. Briefly, low-quality cells (fewer than 500 detected genes and more than 25% mitochondrial reads) were removed from the analysis. The scran, scater and igraph packages were used for basic data analysis, including data normalization, cell cycle annotation, feature selection (of the top 2,000 highly variable genes, excluding genes related to the cell cycle detected using the getVarianceExplained function), principal component analysis (with five significant principal components retained for further analysis using the denoisePCA function) and uniform manifold approximation and projection projection. Barcodes with at least 15 cells in the initial, untreated sample were grouped into three categories: depleted (at least a twofold decrease in the relative frequency); unchanged; and enriched (at least a twofold increase in the relative frequency). The signature score per barcode in a given condition was calculated as the average of the pathway score of individual cells (calculated using the function sumCountsAcrossFeatures from the package scuttle for all cells with the given barcode in the given condition). The significance of the score comparisons between categories was assessed by Wilcoxon rank-sum test (or Kruskal test if there were more than two categories).

### Statistics and reproducibility

The data have been presented as indicated in the figure captions. With the exception of Fig. 1e, the experiments were repeated at least twice with consistent results. Dose–response relationships were analyzed using GraphPad Prism version 9.0 software. The next-generation sequencing experiments were analyzed in R version 4.1.0. No statistical method was used to predetermine sample size but our sample sizes are similar to those reported in previous publications. No data were excluded from the analyses. Unless specified, no assumptions about data distribution were made. The investigators were blinded to the outcome assessment for efficacy studies of the PDX models LU11786 and LU5268.

### Reporting summary

Further information on research design is available in the Nature Portfolio Reporting Summary linked to this article.

## Supplementary information

Supplementary Information Supplementary Tables 1 and 2.

Reporting Summary

Supplementary Data Chemistry and compound note.

## Data Availability

Co-crystal structures that support the findings of this study have been deposited to the Protein Data Bank with the accession numbers 7TYQ, 7TYU and 7TYP and are listed in Supplementary Table 2. Messenger RNA-seq, ATAC-seq and scRNA-seq data that support the findings of this study have been deposited in the Gene Expression Omnibus under accession super series GSE229071. Source data are provided with this paper. All other data supporting the findings of this study are available from the corresponding author upon reasonable request.

## Source data

Source Data Fig. 1 Uncropped western blots.
Source Data Fig. 1 Statistical source data.
Source Data Fig. 2 Statistical source data.
Source Data Fig. 3 Tumor measurement data.
Source Data Fig. 4 Uncropped western blots.
Source Data Fig. 4 Statistical source data.
Source Data Fig. 5 Statistical source data and tumor measurement data.
Source Data Extended Data Fig. 1 Statistical source data.
Source Data Extended Data Fig. 2 Statistical source data.
Source Data Extended Data Fig. 3 Statistical source data.
Source Data Extended Data Fig. 4 Statistical source data.
Source Data Extended Data Fig. 5 Tumor measurement data.
Source Data Extended Data Fig. 6 Statistical source data.
Source Data Extended Data Fig. 7 Tumor measurement data.
Source Data Extended Data Fig. 8 Tumor measurement data.

## Code availability

No custom algorithms were used in this study. Full code will be made available upon request.
