# Supplementary material for: An allosteric pan-TEAD inhibitor blocks oncogenic YAP/TAZ signaling and overcomes KRAS G12C inhibitor resistance
Source: Nat Cancer. 2023 Jun 5;4(6):812–28. doi: 10.1038/s43018-023-00577-0 (PMC10293011; doi:10.1038/s43018-023-00577-0)
Supplement: Supplementary file 1 — Supplementary Tables 1 and 2. [file 43018_2023_577_MOESM1_ESM.pdf]

nature cancer

Article https://doi.org/10.1038/s43018-023-00577-0

An allosteric pan-TEAD inhibitor blocks oncogenic YAP/TAZ signaling and overcomes KRAS G12C inhibitor resistance

In the format provided by the authors and unedited

Supplementary Table S1. Small molecule screening data

Category	Parameter	Description
Assay	Type of assay:	In vitro biochemical binding assay using recombinant proteins
	Target:	YAP_{50-100} (P46937) - TEAD3 (Q99594) PPI.
	Primary measurement:	Detection of TR-FRET signal from the disruption of the YAP-TEAD complex
	Key reagents	His6-TEAD3 (217-447), Genentech<br>Biotin-YAP (50-100), Genentech<br>Streptavidin-XL665 (CIS Bio)<br>Anti-His-europium (PerkinElmer)
	Assay protocol	See Methods “TEAD YAP/TAZ TR-FRET Assay”
	Additional comments	None
Library	Library size	2.2 million compounds
	Library composition	Roche and Genentech internal compound collection. Compounds include internally made molecules for all internal programs and externally sourced compounds.
	Source	Internal and External
	Additional comments	None
Screen	Format	Competitive binding assay with a proximity-based readout of time-resolved fluorescence resonance energy transfer
	Concentration(s) tested	5 uM (2,130,060 compounds), 20 uM (334 compounds), 25 uM (59,156 compounds), 100 uM (5991 compounds). DMSO: 0.5%.
	Plate controls	Neutral control: all reaction components with DMSO as sample<br>Blank: all reaction components except TEAD3 with DMSO as sample<br>Reference inhibitor: peptide 17
	Reagent/ compound dispensing system	Reagent: BioRapTR (Beckman); Compound: Echo 555 acoustic dispenser (Beckman)
	Detection instrument and software	ViewLux (PerkinElmer). Analysis software: Genedata Screener
	Assay validation/QC	RZ’ range: 0.44-0.94, RZ’ average = 0.68.<br>Signal/Background range: 4.0-11.2;<br>Signal/Background average = 6.8
	Correction factors	None
	Normalization	Neutral control – Blank. Analyzed using Genedata Screener software
	Additional comments	
Post-HTS analysis	Hit criteria	Inhibition >/= 20%
	Hit rate	0.085% (1870 of 2,200,000)
	Additional assay(s)	Confirmation in dose-response in YAP/TEAD3 PPI TR-FRET assay; counterscreen in dose-response in TR-FRET assay with 6x-His-Biotin in place of YAP/TEAD3 complex
	Confirmation of hit purity and structure	All hits where resynthesized and compounds tested for purity via NMR and LC-MS.
	Additional comments	Please see chart for HTS workflow.

Supplementary Table S2. X-ray crystallography data collection and refinement statistics

	TEAD2/Compound 1<br>PDB: 7TYQ	TEAD2/Compound 2<br>PDB: 7TYU	TEAD2/GNE-7883<br>PDB: 7TYP
**Data collection**			
Space group	C121	C121	C121
Cell dimensions			
a, *b*, *c* (Å)	114.85, 61.18, 79.95	115.27, 61.53, 79.41	122.97, 61.43, 80.05
α, β, γ (°)	90, 111.61, 90	90, 111.759, 90	90, 117.18, 90
Wavelength (Å)	1.00000	0.97946	0.99999
Resolution (Å)	53.74-1.88 (ellipsoidal)<br>53.74-2.30 (isotropic)<br>(1.91-1.88)*	53.84-1.78 (ellipsoidal)<br>53.84-2.16 (isotropic)<br>(1.82-1.78)	71.21-1.60 (ellipsoidal)<br>71.21-1.90 (isotropic)<br>(1.76-1.60)
R_{merge}	0.059 (0.991)	0.025 (0.576)	0.032 (0.741)
$I / \sigma(I)$	16.2 (1.7)	14.0 (1.5)	17.2 (1.5)
CC(1/2)	0.999 (0.711)	1.00 (0.783)	0.999 (0.652)
Completeness	94.4 (73.1)	92.2 (61.3)	89.2 (68.4)
Redundancy	6.8 (7.2)	3.4 (3.6)	3.3 (3.5)
**Refinement**			
Resolution (Å)	74.33-1.88 (anisotropic)	53.84-1.78 (anisotropic)	71.21-1.60 (anisotropic)
No. reflections	28082 (1405)	31238 (1563)	42783 (2140)
R_{work} / R_{free}	0.2061/0.2472	0.1972/0.2298	0.1840/0.2156
No. atoms			
Protein	3251	3268	3361
Ligand/ion	50	70	90
Water	66	81	190
B-factors			
Protein	49.2	51.9	37.5
Ligand/ion	41.3	45.2	26.7
Water	45.1	48.1	44.0
R.m.s. deviations			
Bond lengths (Å)	0.004	0.004	0.01
Bond angles (°)	0.67	0.71	1.38

*Values in parentheses are for highest-resolution shell.
