# Supplementary material for: An allosteric pan-TEAD inhibitor blocks oncogenic YAP/TAZ signaling and overcomes KRAS G12C inhibitor resistance
Source: Nat Cancer. 2023 Jun 5;4(6):812–28. doi: 10.1038/s43018-023-00577-0 (PMC10293011; doi:10.1038/s43018-023-00577-0)
Supplement: Supplementary file 2 — Reporting Summary [file 43018_2023_577_MOESM2_ESM.pdf]

nature research

Corresponding author(s): Marie Evangelista, Xin Ye, James Crawford, Anwesha Dey

Last updated by author(s): Apr 25, 2023

Reporting Summary

Nature Research wishes to improve the reproducibility of the work that we publish. This form provides structure for consistency and transparency in reporting. For further information on Nature Research policies, see our Editorial Policies and the Editorial Policy Checklist.

Statistics

For all statistical analyses, confirm that the following items are present in the figure legend, table legend, main text, or Methods section.

n/a	Confirmed	
☐	☒	The exact sample size (n) for each experimental group/condition, given as a discrete number and unit of measurement
☐	☒	A statement on whether measurements were taken from distinct samples or whether the same sample was measured repeatedly
☐	☒	The statistical test(s) used AND whether they are one- or two-sided<br>*Only common tests should be described solely by name; describe more complex techniques in the Methods section.*
☐	☒	A description of all covariates tested
☐	☒	A description of any assumptions or corrections, such as tests of normality and adjustment for multiple comparisons
☐	☒	A full description of the statistical parameters including central tendency (e.g. means) or other basic estimates (e.g. regression coefficient) AND variation (e.g. standard deviation) or associated estimates of uncertainty (e.g. confidence intervals)
☐	☒	For null hypothesis testing, the test statistic (e.g. F, t, r) with confidence intervals, effect sizes, degrees of freedom and P value noted<br>*Give P values as exact values whenever suitable.*
☒	☐	For Bayesian analysis, information on the choice of priors and Markov chain Monte Carlo settings
☒	☐	For hierarchical and complex designs, identification of the appropriate level for tests and full reporting of outcomes
☒	☐	Estimates of effect sizes (e.g. Cohen's d, Pearson's r), indicating how they were calculated

Our web collection on statistics for biologists contains articles on many of the points above.

Software and code

Policy information about availability of computer code

Data collection	For cell proliferation assays, data were collected using Tecan; Infinite M1000 Pro or EnVision Manager 1.14.3049.1193. For immuno staining, data was collected using Harmony 4.9 software, for Western blot, images were acquired using LiCOR Odyssey CLx. Cell growth curves were collected using Incucyte software v. 2018A
Data analysis	ATAC-seq reads were analyzed using the ENCODE ATAC-seq pipeline v1. Briefly, reads were trimmed of adapters by cutadapt (version 1.9.1) (10) and mapped to hg38 by Bowtie2 (version 2.2.6) (11). Bam files were converted to tagAlign format, which was then adjusted for Tn5 by shifting +4 bp for positive strand and by −5bp for negative strand. TagAlign files were used to call peaks using MACS2 (version 2.1.0) (12) and those peaks with p<1e-6 were retained for differential analysis by Diffbind (version 3.0.13) (13) in figure 1 or the bamCount function of the R package bamsignals (v1.24.0) for figure 3. For RNA-Seq data analyses, RNA-Seq reads were first aligned to ribosomal RNA sequences to remove ribosomal reads. The remaining reads were aligned to the mouse reference genome (GRCm38) using GSNAP version 2013-10-10. scRNA-Seq FASTQ files were processed using kallisto (v. 0.46.2) and bustools (v. 0.40.0) workflow. Downstream analysis of the results was performed in the R environment (R version 4.1, Bioconductor version 3.13), following the OSCA book recommendations. The motif enrichment analysis was performed using either HOMER (version 4.10) with all peaks as the background for Figure 1 or AME (v5.4.1) tool of the MEME suite using the non-differential ATAC-seq peaks as background and HOCOMOCOv11_core_HUMAN motif database.

For manuscripts utilizing custom algorithms or software that are central to the research but not yet described in published literature, software must be made available to editors and reviewers. We strongly encourage code deposition in a community repository (e.g. GitHub). See the Nature Research guidelines for submitting code & software for further information.

Data

Policy information about availability of data

All manuscripts must include a data availability statement. This statement should provide the following information, where applicable:

- Accession codes, unique identifiers, or web links for publicly available datasets
- A list of figures that have associated raw data
- A description of any restrictions on data availability

Crystal structures were deposited to PDB with accession numbers 7TYQ, 7TYU, 7TYP; mRNA-, ATAC- and scRNA-sequencing data that support the findings of this study have been deposited in the Gene Expression Omnibus (GEO) under accession super series GSE229071.

Field-specific reporting

Please select the one below that is the best fit for your research. If you are not sure, read the appropriate sections before making your selection.

☒ Life sciences ☐ Behavioural & social sciences ☐ Ecological, evolutionary & environmental sciences

For a reference copy of the document with all sections, see nature.com/documents/nr-reporting-summary-flat.pdf

Life sciences study design

All studies must disclose on these points even when the disclosure is negative.

Sample size	Typically our efficacy studies vary between 5 to 10 mice. We rely on historical studies (Nat Med . 2015 May;21(5):431-9. doi: 10.1038/nm.3853.); our models have been run numerous times before and we have a full understanding of their performance. In addition, we have done dose escalation studies of the compounds used in this manuscript and we have a good understanding of their effects.
Data exclusions	No data were excluded
Replication	Each experiment were conducted at least twice with consistent results to ensure repeatability of data.
Randomization	Mice bearing tumors are evenly distributed into study groups based on the mean tumor volume of the whole cohort so that the standard deviation is equal across all groups.
Blinding	Blinding was performed for xenograft studies with models LU11788 and LU5268. No blinding were performed for the other experiments. It is not logistically feasible to be truly blinded for in house- studies and It is prohibitive cost-wise. Multiple people are involved in running these studies and everyone is working with integrity and honesty. In addition these models and molecules have been tested multiple times by various researchers in house.

Reporting for specific materials, systems and methods

We require information from authors about some types of materials, experimental systems and methods used in many studies. Here, indicate whether each material, system or method listed is relevant to your study. If you are not sure if a list item applies to your research, read the appropriate section before selecting a response.

Materials & experimental systems

n/a	Involved in the study	
☐	☒	Antibodies
☐	☒	Eukaryotic cell lines
☒	☐	Palaeontology and archaeology
☐	☒	Animals and other organisms
☒	☐	Human research participants
☒	☐	Clinical data
☒	☐	Dual use research of concern

Methods

n/a	Involved in the study	
☒	☐	ChIP-seq
☒	☐	Flow cytometry
☒	☐	MRI-based neuroimaging

Antibodies

Antibodies used	Antibodies used in this study include pan-TEAD (13295, CST, Lot#4) 1:500; YAP (14074, CST, Lot#4) 1:500; TAZ (70148, CST, Lot#1) 1:500; YAP/TAZ (8418, CST, Lot#4) 1:500; MAX (10426-1-AP, Proteintech, lot # 41206) 1:500; α-Tubulin (3873, CST, lot #16) 1:10,000; β-Actin (4970, CST, lot#5) 1:10,000; cleaved PARP (9541, CST Lot #21) 1:3000; p21 (2947, CST, Lot#12) 1:500; anti-rabbit and anti-mouse HRP linked (7074 Lot # 31 and 7076 Lot #38, CST) 1:20,000, IRDye anti-rabbit and anti-mouse (68070 lot #D10831-15 and 68022 lot #D10413-15, LI-COR) 1:20,000; KRAS Rb pAb (12063-1-AP, Proteinech, lot # 112736) 1:1000; Phospho-S6 Ribosomal Protein (Ser235/236) (2211, CST, lot # 23) 1:1000; S6 Ribosomal Protein (5G10) Rabbit mAb (2217, CST, lot # 10) 1:1000; Phospho-MEK1/2 (Ser217/221) (41G9) Rabbit mAb (9154, CST, lot #22) 1:1000; Phospho-p44/42 MAPK (Erk1/2) (Thr202/Tyr204) (D13.14.4E)

XP® Rabbit mAb (4370, CST, lot # 24) 1:1000; p44/42 MAPK (Erk1/2) (3A7) Mouse mAb (9107, CST, lot #10) 1:1000; Phospho-p90RSK (Ser380) (D5D8) Rabbit mAb (12032, CST, lot #1) 1:1000; Purified Mouse Anti-Rsk Clone 78/RSK (610226, BD Biosciences, lot # 9147501) 1:1000; Vimentin (D21H3) Mouse mAb (5741, CST, Lot #5) 1:1000; Mouse Anti-MEK1 Clone 25/MEK1 (610122, BD Biosciences, lot #8337758) 1:1000; Phospho-Akt (Ser473) (D9E) XP® Rabbit mAb (4060, CST, lot#27) 1:1000; Akt (pan) (40D4) Mouse mAb (2920, CST, lot #8) 1:1000.

Validation

Antibodies are from commercial sources and validation can be found on the product websites.

Eukaryotic cell lines

Policy information about cell lines

Cell line source(s)

Cell lines used in this study were obtained from Genentech cell line bank (Nature 2015 Apr 16;520(7547):307-11.) or American Type Culture Collection (ATCC): OVCAR-8 (RRID:CVCL_1629), HCC1576 (RRID:CVCL_V597), MDA-MB-231 (ATCC HTB-26), NCI-H226 (ATCC CRL-5826), Detroit562 (ATCC CCL-138), PATU-8988-T (RRID:CVCL_1847), SK-N-FI (ATCC CRL-2142), NCI-H290 (RRID:CVCL_A555), NCI-H2591 (RRID:CVCL_A543), NCI-H2373 (RRID:CVCL_A533), NCI-H2369 (RRID:CVCL_A532), NCI-H2803 (RRID:CVCL_U997), NCI-H2804 (RRID:CVCL_U998), NCI-H2722 (RRID:CVCL_U994), NCI-H2691 (RRID:CVCL_A551), NCI-H2596 (RRID:CVCL_A546), JL-1 (RRID:CVCL_2080), MSTO-211H (ATCC CRL-2081), NCI-H23 (ATCC CRL-5800), NCI-H358 (ATCC CRL-5807), SW837 (ATCC CCL-235)

Authentication

Cell line authentication was routinely conducted by SNP-based genotyping using Fluidigm multiplexed assays at the Genentech cell line core facility.

Mycoplasma contamination

All cell lines used in this study tested negative for mycoplasma contamination.

Commonly misidentified lines
(See ICLAC register)

N/A

Animals and other organisms

Policy information about studies involving animals; ARRIVE guidelines recommended for reporting animal research

Laboratory animals

Female C.B-17 SCID (Inbred) were obtained from Charles River-Hollister. Genentech is an AAALAC-accredited facility and all animal activities in this research study were conducted under protocols approved by the Genentech Institutional Animal Care and Use Committee (IACUC). The maximal tumor size permitted by the ethics committee/institutional review board is 2 cm3. The maximal tumor size/burden was never exceeded in the studies. Source data is provided for all in vivo experiments. Mice were fed ad libitum with an autoclaved rodent diet (LabDiet 5010).Mice were housed in individually ventilated cages within animal rooms maintained on a 14:10-hour, light:dark cycle. Animal rooms were temperature and humidity-controlled, between 68 to 79°F (20.0 to 26.1°C) and 30 to 70% respectively, with 10 to 15 room air exchanges per hour. All mice used in the study are females that are 7-10 weeks of age at the start of the study. Animals were maintained in accordance with the Guide for the Care and Use of Laboratory Animals (National Research Council 2011).

Wild animals

N/A

Field-collected samples

N/A

Ethics oversight

The animal study protocols have been approved by the Genentech Institutional Animal Care and Use Committee.

Note that full information on the approval of the study protocol must also be provided in the manuscript.
