# Supplementary material for: An allosteric pan-TEAD inhibitor blocks oncogenic YAP/TAZ signaling and overcomes KRAS G12C inhibitor resistance
Source: Nat Cancer. 2023 Jun 5;4(6):812–28. doi: 10.1038/s43018-023-00577-0 (PMC10293011; doi:10.1038/s43018-023-00577-0)

Synthetic procedures for TEAD Small Molecule Inhibitors (SMI)

Intermediate 1

Ethyl 5-chloro-7-oxo-4,7-dihydropyrazolo[1,5-*a*]pyrimidine-3-carboxylate

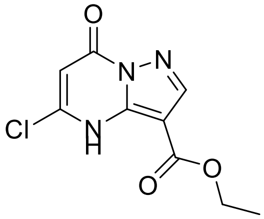

Step 1: Ethyl 5,7-dioxo-4,5,6,7-tetrahydropyrazolo[1,5-*a*]pyrimidine-3-carboxylate

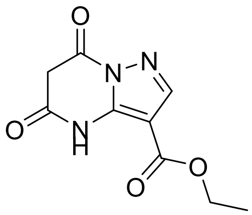

To a stirred mixture of sodium *tert*-butoxide (46.5 g, 483.4 mmol) in ethanol (400 mL) at 0 °C was added ethyl 5-amino-1*H*-pyrazole-4-carboxylate (25.0 g, 161.1 mmol) and diethyl malonate (77.4 g, 483.4 mmol), and the reaction mixture was stirred at 50 °C for 16 hours. The cooled reaction mixture was quenched with water (300 mL) and then adjusted to pH 6 with 1 N HCl. The resulting precipitate was collected by filtration and dried under vacuum to afford ethyl 5,7-dioxo-4,5,6,7-tetrahydropyrazolo[1,5-*a*]pyrimidine-3-carboxylate (30.0 g, 83%) as a white solid. ^{1}H NMR (400 MHz, DMSO-d_6): δ 9.04 (s, 1H), 7.78 (s, 1H), 4.26-4.20 (m, 4H), 1.27 (t, J = 7.2 Hz, 3H); LCMS (ESI): m/z 223.9 $(M+H)^+$.

Step 2: Ethyl 5,7-dichloropyrazolo[1,5-*a*]pyrimidine-3-carboxylate

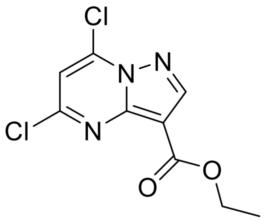

A mixture of ethyl 5,7-dioxo-4,5,6,7-tetrahydropyrazolo[1,5-*a*]pyrimidine-3-carboxylate (20.0 g, 89.61 mmol) and phosphoryl chloride (43.21 mL, 465.0 mmol) was stirred at 100 °C for 16 hours. The reaction mixture was slowly quenched with water (100 mL) and saturated aq. sodium bicarbonate solution was added dropwise to adjust the reaction to pH 7. The mixture was then extracted with ethyl acetate (300 mL × 2). The combined organic layers were dried over

anhydrous sodium sulfate, filtered, and concentrated under reduced pressure to afford ethyl 5,7-dichloropyrazolo[1,5-*a*]pyrimidine-3-carboxylate (5.5 g, 24%) as a yellow solid.

Step 3: Ethyl 5-chloro-7-oxo-4,7-dihydropyrazolo[1,5-*a*]pyrimidine-3-carboxylate

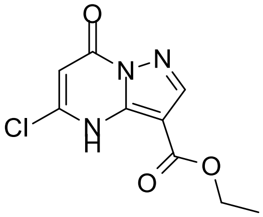

To a stirred solution of ethyl 5,7-dichloropyrazolo[1,5-*a*]pyrimidine-3-carboxylate (2.0 g, 7.69 mmol) in tetrahydrofuran (12 mL) was added 2M NaOH (9.61 mL, 19.22 mmol), and the reaction mixture was stirred at room temperature for 1 hour. Volatile solvent was removed, and the remaining aqueous mixture was quenched with saturated aq. ammonium chloride solution (50 mL). The reaction mixture was extracted with ethyl acetate (300 mL × 2). The combined organic layers were dried over anhydrous magnesium sulfate, filtered, and concentrated under reduced pressure to afford the title compound (1.5 g, 81%). ^{1}H NMR (400 MHz, DMSO-d_6): δ 8.03 (s, 1H), 5.58 (s, 1H), 4.18 (q, J = 7.2 Hz, 2H), 1.25 (t, J = 7.2 Hz, 3H); LCMS (ESI): *m/z* 242.1 $(M+H)^+$.

Intermediate 2

5-(4-Cyclohexylphenyl)-7-oxo-4,7-dihydropyrazolo[1,5-*a*]pyrimidine-3-carboxylic acid

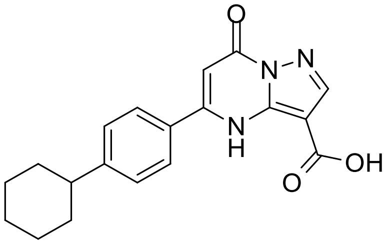

Step 1: Ethyl 3-(4-cyclohexylphenyl)-3-oxopropanoate

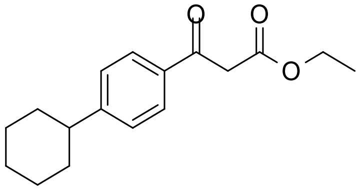

To a stirred solution of 1-(4-cyclohexylphenyl)ethanone (40.0 g, 197.0 mmol) in tetrahydrofuran (400 mL) was added sodium hydride (17.4 g, 435 mmol, 60% in mineral oil) portion wise at 0 ºC. After stirring at 0 ºC for 1 hour, diethyl carbonate (70.0 g, 593.0 mmol) was added dropwise, and the reaction mixture was stirred at ambient temperature for 5 hours. The reaction mixture

was quenched with saturated aq. ammonium chloride solution (500 mL) and extracted with ethyl acetate (500 mL × 2). The combined organic layers were washed with brine (500 mL × 2), filtered, and concentrated under reduced pressure. The crude residue was purified by column chromatography on silica gel (0-10% ethyl acetate in petroleum ether) to afford ethyl 3-(4-cyclohexylphenyl)-3-oxopropanoate (50.0 g, 92%, ketone/enol = 2.4:1) as a yellow oil. ^{1}H NMR (400 MHz, $CDCl_3$): *δ* 12.58 (s, 1H of enol), 7.86 (d, *J* = 8.4 Hz, 2H of ketone), 7.71 (d, *J* = 8.4 Hz, 2H of enol), 7.32 (d, *J* = 8.4 Hz, 2H of ketone), 7.26 (d, *J* = 8.4 Hz, 2H of enol), 5.64 (s, 1H of enol), 4.25-4.17 (m, 4H), 3.92 (s, 2H of ketone), 2.59-2.51 (m, 2H), 1.80-1.77 (m, 8H), 1.71-1.68 (m, 2H), 1.45-1.41 (m, 8H), 1.34 (t, *J* = 7.2 Hz, 3H of enol), 1.32-1.29 (m, 2H), 1.27 (t, *J* = 7.2 Hz, 3H of ketone).

Step 2: Ethyl 5-(4-cyclohexylphenyl)-7-oxo-4,7-dihydropyrazolo[1,5-*a*]pyrimidine-3-carboxylate

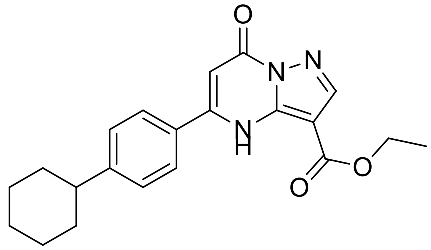

A mixture of ethyl 3-(4-cyclohexylphenyl)-3-oxopropanoate (29.5 g, 107 mmol), *p*-toluenesulfonic acid monohydrate (2.0 g, 9.0 mmol) and ethyl 5-amino-1*H*-pyrazole-4-carboxylate (13.0 g, 84 mmol) in *n*-butanol (50 mL) was stirred at 130 °C for 16 hours. The reaction mixture was cooled to room temperature and then poured into petroleum ether (1 L). The resulting solid was filtered and dried under vacuum to afford ethyl 5-(4-cyclohexylphenyl)-7-oxo-4,7-dihydropyrazolo[1,5-a]pyrimidine-3-carboxylate (26.0 g, 85%) as a yellow solid. ^{1}H NMR (400 MHz, DMSO-d_6): *δ* 11.59 (s, 1H), 8.25 (s, 1H), 7.69 (d, *J* = 8.4 Hz, 2H), 7.43 (d, *J* = 8.4 Hz, 2H), 6.24 (s, 1H), 4.31 (q, *J* = 7.2 Hz, 2H), 2.65-2.56 (m, 1H), 1.82-1.77 (m, 4H), 1.74-1.68 (m, 1H), 1.48-1.38 (m, 4H), 1.36 (t, *J* = 7.2 Hz, 3H), 1.28-1.25 (m, 1H); LCMS (ESI): *m/z* 366.2 $(M+H)^+$.

Step 3: 5-(4-Cyclohexylphenyl)-7-oxo-4,7-dihydropyrazolo[1,5-*a*]pyrimidine-3-carboxylic acid

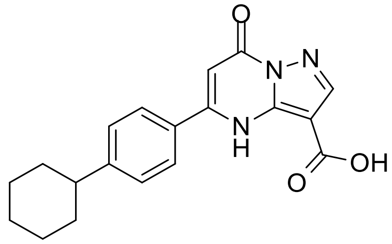

A mixture of lithium hydroxide monohydrate (30.0 g, 712.0 mmol) and ethyl 5-(4-cyclohexylphenyl)-7-oxo-4,7-dihydropyrazolo[1,5-*a*]pyrimidine-3-carboxylate (26.0 g, 71.0 mmol) in water (100 mL) and ethanol (100 mL) was stirred at 80 °C for 16 hours. Volatile solvent was removed under reduced pressure. The aqueous crude residue was adjusted to pH 6 using concentrated aq. HCl. The resulting precipitate was filtered and dried under vacuum to afford the title compound (20.0 g, 83%) as a white solid. ^{1}H NMR (400 MHz, DMSO-d_6): δ 8.19 (s, 1H), 7.71 (d, J = 8.0 Hz, 2H), 7.39 (d, J = 8.0 Hz, 2H), 6.21 (s, 1H), 2.65-2.55 (m, 1H), 1.85-1.74 (m, 4H), 1.72-1.66 (m, 1H), 1.49-1.30 (m, 4H), 1.29-1.17 (m, 1H); LCMS (ESI): *m/z* 338.2 $(M+H)^+$.

Compound 1

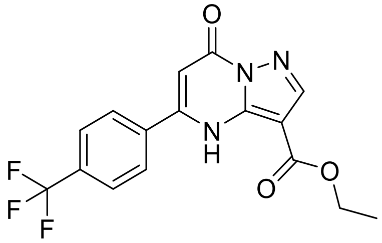

Step 1: Ethyl 3-oxo-3-(4-(trifluoromethyl)phenyl)propanoate

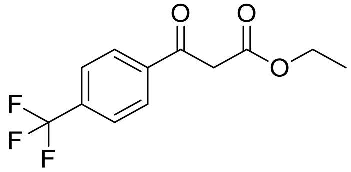

To a stirred solution of 1-(4-(trifluoromethyl)phenyl)ethanone (3.88 g, 20.62 mmol) in toluene (60 mL) at 0 °C was added sodium hydride (2.31 g, 57.74 mmol, 60% in mineral oil). After 30 minutes, a solution of diethyl carbonate (7.31 g, 61.87 mmol) in toluene (5 mL) was added dropwise under N_2 atmosphere at 0 °C. The resulting reaction mixture was stirred at 100 °C for 1 hour. The solution was cooled to room temperature and quenched with water (60 mL × 2). The aqueous phase was extracted with ethyl acetate (400 mL × 2). The combined organic layers were washed with brine, dried over anhydrous sodium sulfate, filtered, and concentrated under reduced pressure. The crude residue was purified by column chromatography on silica gel (0-3% ethyl acetate in petroleum ether) to afford ethyl 3-oxo-3-(4-(trifluoromethyl)phenyl)propanoate (3.2 g, 39%) as light yellow oil. LCMS (ESI): *m/z* 260.9 $(M+H)^+$.

Step 2: Ethyl 7-oxo-5-(4-(trifluoromethyl)phenyl)-4,7-dihydropyrazolo[1,5-*a*]pyrimidine-3-carboxylate

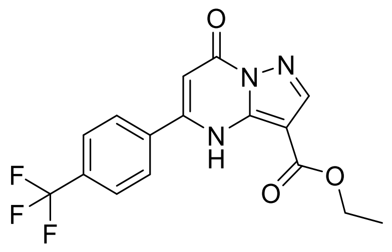

A mixture of ethyl 3-oxo-3-(4-(trifluoromethyl)phenyl)propanoate (200 mg, 0.77 mmol) and ethyl 5-amino-1*H*-pyrazole-4-carboxylate (143 mg, 0.92 mmol) in concentrated acetic acid (2 mL) was stirred at 110 °C for 5 hours. The solution was cooled to room temperature and adjusted to pH 8 with saturated aq. sodium bicarbonate solution. The reaction mixture was then extracted with dichloromethane (10 mL × 3). The combined organic layers were dried over anhydrous sodium sulfate, filtered, and concentrated under reduced pressure. The crude residue was purified by prep-HPLC [Agela, DuraShell 150mm*25mm*5um, water (10 mM NH_4HCO_3)-Acetonitrile, 50-80%] to afford the title compound (42 mg, 15%) as a white solid. ^{1}H NMR (400 MHz, DMSO) δ 11.96 (br s, 1H), 8.26 (d, *J* = 8.3 Hz, 2H), 8.13 (s, 1H), 7.83 (d, *J* = 8.4 Hz, 2H), 6.32 (s, 1H), 4.23 (q, *J* = 7.1 Hz, 2H), 1.32 (t, *J* = 7.1 Hz, 3H). ^{13}C NMR (101 MHz, DMSO) δ 162.91, 158.16, 155.71, 150.23, 144.63, 142.55, 129.75 (q, *J* = 32 Hz, <u>C</u>-CF_3), 128.22, 125.80-125.56 (m, -<u>C</u>HC-CF_3), 124.79 (q, *J* = 272.7 Hz, <u>C</u>F_3), 98.70, 94.03, 59.09, 15.02. ^{19}F NMR (376 MHz, DMSO) δ -60.98. HRMS (ESI+) m/z found MH^+ 352.0902, $C_{16}H_{13}F_3N_3O_3$ requires 352.0909. FTIR (KBr pellet): V_{max} 3519 (N-H stretch), 3199 (CH stretch alkene), 2985 (CH stretch alkane), 1671 (CO stretch), 1626 (C=C stretch), 1559 (C=C stretch).

Compound 2

(*R*)-1-(5-(4-Cyclohexylphenyl)-7-oxo-4,7-dihydropyrazolo[1,5-*a*]pyrimidine-3-carbonyl)pyrrolidine-3-carbonitrile

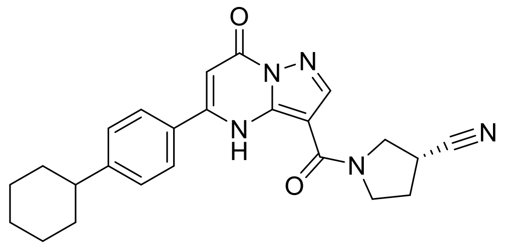

To a stirred solution of 5-(4-cyclohexylphenyl)-7-oxo-4,7-dihydropyrazolo[1,5-*a*]pyrimidine-3-carboxylic acid (Intermediate 2, 10.0 g, 29.64 mmol) and *N*,*N*-diisopropylethylamine (24.5 mL, 148.2 mmol) in dichloromethane (300 mL) at room temperature was added (1-

[bis(dimethylamino)methylene]-1*H*-1,2,3-triazolo[4,5-*b*]pyridinium 3-oxide hexafluorophosphate (18.03 g, 47.43 mmol), and the reaction mixture was stirred for 20 minutes. (3*R*)-pyrrolidine-3-carbonitrile hydrochloride (5.9 g, 44.46 mmol) was then added, and the reaction mixture was stirred for 2 hours. The reaction mixture was diluted with ethyl acetate (1 L) and washed with 10% aq. citric acid solution (100 mL × 3). The organic layer was dried over anhydrous sodium sulfate, filtered, and concentrated under reduced pressure. Methanol (300 mL) was added, and the resulting precipitate was filtered and dried under vacuum to afford 12 g of crude product. The crude product was purified by column chromatography on silica gel (0-5% methanol in dichloromethane) to afford the title compound (9.46 g, 77%) as a white solid. ^{1}H NMR (400 MHz, DMSO-d_6) δ 11.37 (br s, 1H), 8.31 (s, 1H), 7.78-7.71 (m, 2H), 7.51-7.43 (m, 2H), 6.28 (s, 1H), 4.10-3.95 (m, 1H), 3.95-3.70 (m, 3H), 3.60-3.50 (m, 1H), 2.71-2.58 (m, 1H), 2.46-2.35 (m, 1H), 2.33-2.21 (m, 1H), 1.93-1.79 (m, 4H), 1.79-1.70 (m, 1H), 1.55-1.35 (m, 4H), 1.35-1.22 (m, 1H). ^{13}C NMR (101 MHz, DMSO-d_6, 360°K) δ 162.47, 156.03, 151.96, 149.81, 144.94, 141.82, 129.54, 128.19, 127.11, 121.10, 99.72, 96.56, 50.02, 46.08, 44.07, 34.09, 29.51, 27.68, 26.68, 25.99. HRMS (ESI+) m/z found MH^+ 416.2078, $C_{24}H_{26}N_5O_2$ requires 416.2087. FTIR (KBr pellet): V_{max} 3461 (NH stretch), 3085 (CH stretch alkene), 2923 (CH stretch alkane), 2247 (CN stretch), 1704 (CO stretch), 1612 (C=C stretch), 1473 (CH stretch cyclic alkane).

GNE-7883

5-(4-Cyclohexylphenyl)-3-(3-(fluoromethyl)azetidine-1-carbonyl)-2-(3-methylpyrazin-2-yl)pyrazolo[1,5-*a*]pyrimidin-7(4*H*)-one

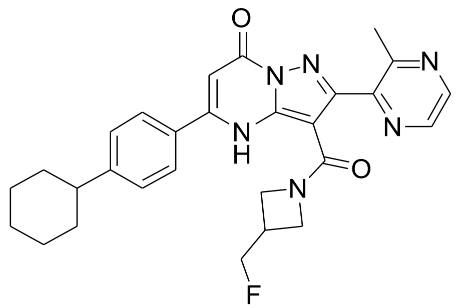

Step 1: 2-Methyl-3-(tributylstannyl)pyrazine

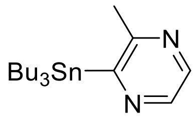

To a stirred solution of 2-bromo-3-methyl-pyrazine (1.0 g, 5.78 mmol) and tributyltin chloride (2.41 g, 7.40 mmol) in tetrahydrofuran (4 mL) at -78 °C under a N_2 atmosphere was added *n*-butyllithium (2.8 mL, 7 mmol, 2.5M in hexane) dropwise. The reaction mixture was stirred for 2 hours at -78 °C and then quenched with water (50 mL). The mixture was extracted with hexane (50 mL × 2), and the combined organic layers were dried over anhydrous sodium sulfate, filtered, and concentrated under reduced pressure. The crude residue was purified by column chromatography on silica gel (0-10% ethyl acetate in petroleum ether) to afford 2-methyl-3-(tributylstannyl)pyrazine (800 mg, 36%) as a colorless oil. ^{1}H NMR (400 MHz, $CDCl_3$): δ 8.48 (d, J = 2.4 Hz, 1H), 8.25 (d, J = 2.4 Hz, 1H), 2.56 (s, 3H), 1.58-1.45 (m, 6H), 1.34-1.27 (m, 6H), 1.19-1.08 (m, 6H), 0.90-0.81 (m, 9H).

Step 2: 5-(4-Cyclohexylphenyl)-3-(3-(fluoromethyl)azetidine-1-carbonyl)-2-(3-methylpyrazin-2-yl)pyrazolo[1,5-*a*]pyrimidin-7(4*H*)-one

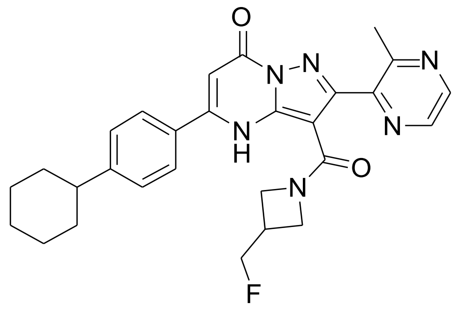

In a microwave vial was placed 2-bromo-5-(4-cyclohexylphenyl)-3-(3-(fluoromethyl)azetidine-1-carbonyl)pyrazolo[1,5-*a*]pyrimidin-7(4*H*)-one (100 mg, 0.21 mmol, produced in analogy to intermediate 2 and compound 2), 2-methyl-3-(tributylstannyl)pyrazine (157 mg, 0.41 mmol), cesium fluoride (94 mg, 0.62 mmol), copper(I) chloride (3 mg, 0.03 mmol), and (1,1'-bis(diphenylphosphino)ferrocene)palladium(II) dichloride (15 mg, 0.02 mmol) in *N,N*-dimethylacetamide (4 mL). The vial was capped, and the reaction mixture was irradiated by microwave at 140 °C. The reaction mixture was quenched with 5% aq. citric acid solution (20 mL) and extracted with ethyl acetate (50 mL × 3). The combined organic layers were dried over anhydrous sodium sulfate, filtered, and concentrated under reduced pressure. The crude residue was purified by prep-TLC (10 % methanol in dichloromethane) to afford the title compound (86 mg, 84%) as a white solid. ^{1}H NMR (400 MHz, DMSO) δ 12.30 (br s, 1H), 8.67-8.58 (m, 2H), 7.78-7.70 (m, 2H), 7.48-7.43 (m, 2H), 6.19 (s, 1H), 4.55 (d, J = 5.9 Hz, 1H), 4.43 (d, J = 5.8 Hz, 1H), 4.20-3.33 (m, 4H), 2.96-2.79 (m, 1H), 2.68 (s, 3H), 2.67-2.57 (m, 1H), 1.87-1.77 (m, 4H), 1.77-1.68 (m, 1H), 1.57-1.33 (m, 4H), 1.33-1.19 (m, 1H). ^{13}C NMR (101 MHz,

DMSO, 360°K) δ 163.91, 155.98, 153.26, 151.61, 151.57, 150.52, 146.48, 144.12, 142.17, 141.89, 130.30, 128.01, 127.76, 99.53, 96.28, 85.24, 83.60, 51.16 (d, *J* = 9.1 Hz), 44.11, 34.16, 29.32, 29.11, 29.22 (d, *J* = 21.2 Hz), 26.71, 26.01, 22.66. ^{19}F NMR (376 MHz, DMSO) δ -224.70. HRMS (ESI+) m/z found MH^+ 501.2411, $C_{28}H_{30}FN_6O_2$ requires 501.2414. FTIR (KBr pellet): V_{max} 3438 (NH stretch), 2925 (CH stretch alkane), 2851 (CH-stretch alkane), 1680 (CO stretch), 1614 (C=C stretch), 1426 (CH stretch cyclic alkane).

Compound 3

5-(4-Cyclohexylphenyl)-3-(3-(fluoromethyl)azetidine-1-carbonyl)-2-(pyrazin-2-yl)pyrazolo[1,5-*a*]pyrimidin-7(4*H*)-one

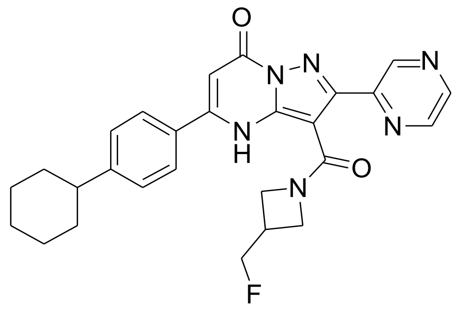

Similar to the procedure described for GNE-7883, replacing 2-bromo-3-methyl-pyrazine with 2-bromopyrazine, 5-(4-cyclohexylphenyl)-3-(3-(fluoromethyl)azetidine-1-carbonyl)-2-(pyrazin-2-yl)pyrazolo[1,5-*a*]pyrimidin-7(4*H*)-one was obtained as a white solid. ^{1}H NMR (400 MHz, DMSO) *δ* 12.60 (s, 1H), 9.22 (s, 1H), 8.80-8.70 (m, 2H), 7.72 (d, *J* = 7.8 Hz, 2H), 7.44 (d, *J* = 7.8 Hz, 2H), 6.15 (s, 1H), 4.60 (d, *J* = 5.9 Hz, 1H), 4.49 (d, *J* = 5.9 Hz, 1H), 4.22-3.98 (br s, 1H), 3.98-3.70 (br s, 2H), 3.67-3.44 (br s, 1H), 3.00-2.83 (m, 1H), 2.68-2.56 (m, 1H), 1.82 (d, *J* = 10.9 Hz, 4H), 1.77-1.67 (m, 1H), 1.54-1.19 (m, 5H). ^{13}C NMR (101 MHz, DMSO, 360°K) *δ* 163.35, 156.02, 151.96, 151.54, 148.71, 147.45, 145.07, 144.75, 143.63, 141.85, 130.40, 128.23, 127.65, 98.89, 96.04, 85.38, 83.74, 51.14, 44.13, 34.18, 29.10 (d, *J* = 21.2 Hz), 26.72, 26.02. ^{19}F NMR (376 MHz, DMSO) *δ* -223.79. HRMS (ESI+) m/z found MH^+ 487.2253, $C_{27}H_{28}FN_6O_2$ requires 487.2258. FTIR (KBr pellet): V_{max} 3452 (NH stretch), 2925 (CH-stretch alkane), 2853 (CH-stretch alkane), 1698 (CO stretch), 1620 (C=C stretch), 1392 (CH stretch cyclic alkane).

Compound 4

Ethyl 5-(4-(benzyl(methyl)amino)phenyl)-7-oxo-4,7-dihydropyrazolo[1,5-*a*]pyrimidine-3-carboxylate

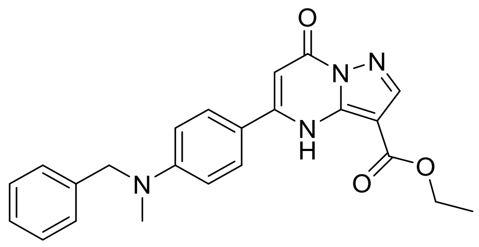

Step 1: *N*-Benzyl-4-bromo-*N*-methylaniline

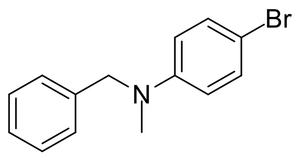

A mixture of 4-bromo-*N*-methylaniline (500 mg, 2.69 mmol), benzaldehyde (371 mg, 3.49 mmol) and sodium triacetoxyborohydride (1.71 g, 8.06 mmol) in 1,2-dichloroethane (15 mL) was stirred at room temperature for 16 hours. The reaction mixture was diluted with water (20 mL) and extracted with dichloromethane (20 mL × 2). The combined organic layers were dried over anhydrous sodium sulfate, filtered, and concentrated under reduced pressure. The crude residue was purified by column chromatography on silica gel (0-5% ethyl acetate in petroleum ether) to afford *N*-benzyl-4-bromo-*N*-methylaniline (700 mg, 94%) as a yellow oil. LCMS (ESI): *m/z* 275.9 $(M+H)^+$.

Step 2: *N*-Benzyl-*N*-methyl-4-(4,4,5,5-tetramethyl-1,3,2-dioxaborolan-2-yl)aniline

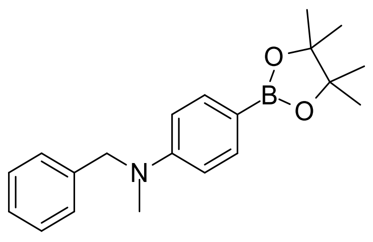

To a stirred mixture of *N*-benzyl-4-bromo-*N*-methylaniline (700 mg, 2.53 mmol), KOAc (747 mg, 7.60 mmol), 4,4,4',4',5,5,5',5'-octamethyl-2,2'-bi(1,3,2-dioxaborolane) (1.29 g, 5.07 mmol) in 1,4-dioxane (30 mL) was added (1,1'-bis(diphenylphosphino)ferrocene)palladium(II) dichloride (186 mg, 0.25 mmol). The reaction mixture was stirred at 80 °C for 16 hours under N_2 atmosphere. The cooled reaction was quenched with water (40 mL) and then extracted with ethyl acetate (40 mL × 2). The combined organic layers were washed with brine (20 mL × 2), dried over anhydrous sodium sulfate, filtered, and concentrated under reduced pressure.

The crude residue was purified by column chromatography on silica gel (0-10% ethyl acetate in petroleum ether) to afford *N*-benzyl-*N*-methyl-4-(4,4,5,5-tetramethyl-1,3,2-dioxaborolan-2-yl)aniline (420 mg, 35%) as a yellow solid. ^{1}H NMR (400 MHz, $CDCl_3$): *δ* 7.68 (d, *J* = 8.8 Hz, 2 H), 7.35-7.28 (m, 2 H), 7.25-7.18 (m, 3 H), 6.74 (d, *J* = 8.8 Hz, 2 H), 4.59 (s, 2 H), 3.06 (s, 3 H), 1.32 (s, 12 H); LCMS (ESI): *m/z* 324.0 $(M+H)^{+}$.

Step 3: Ethyl 5-(4-(benzyl(methyl)amino)phenyl)-7-oxo-4,7-dihydropyrazolo[1,5-*a*]pyrimidine-3-carboxylate

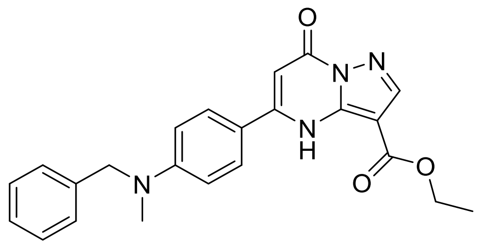

A mixture of ethyl 5-chloro-7-oxo-4,7-dihydropyrazolo[1,5-*a*]pyrimidine-3-carboxylate (Intermediate 1, 100 mg, 0.30 mmol), *N*-benzyl-*N*-methyl-4-(4,4,5,5-tetramethyl-1,3,2-dioxaborolan-2-yl)aniline (284 mg, 0.60 mmol), (1,1'-bis(diphenylphosphino)ferrocene)palladium(II) dichloride (22 mg, 0.03 mmol), and sodium carbonate (96 mg, 0.91 mmol) in 1,4-dioxane (10 mL) and water (2 mL) was stirred at 100 °C for 16 hours under a N_2 atmosphere. The cooled reaction mixture was quenched with water (30 mL) and extracted with ethyl acetate (30 mL × 2). The combined organic layers were washed with brine (60 mL), dried with anhydrous sodium sulfate, filtered, and concentrated under reduced pressure. The crude residue was purified by prep-TLC (0-5% methyl alcohol in dichloromethane) to afford the title compound (19 mg, 15%) as a white solid. ^{1}H NMR (400 MHz, DMSO) *δ* 11.11 (s, 1H), 8.20 (s, 1H), 7.68-7.59 (m, 2H), 7.37-7.70 (m, 2H), 7.27-7.23 (m, 1H), 7.23-7.18 (m, 2H), 6.92-6.83 (m, 2H), 6.17 (s, 1H), 4.72 (s, 2H), 4.30 (q, *J* = 7.0 Hz, 2H), 3.15 (s, 3H), 1.34 (t, *J* = 7.0 Hz, 3H). ^{13}C NMR (101 MHz, DMSO) δ 162.21, 156.11, 151.76, 151.20, 143.90, 143.39, 138.74, 129.04, 129.00, 127.33, 127.01, 118.15, 112.17, 97.72, 94.62, 60.44, 55.28, 39.30, 14.78. HRMS (ESI+) m/z found MH^{+} 403.1762, $C_{23}H_{23}N_4O_3$ requires 403.1770. FTIR (KBr pellet): V_{max} 3352 (N-H stretch), 1692 (CO stretch), 1622 (C=C stretch).

Compound 1

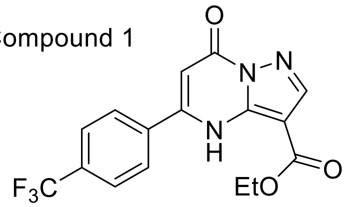

GNE-7883

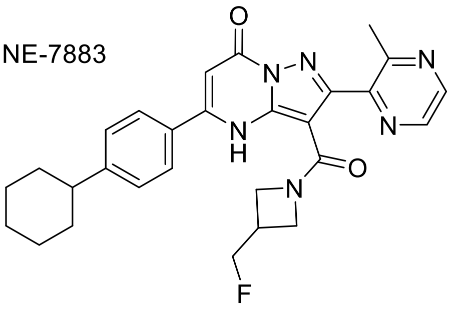

Compound 2

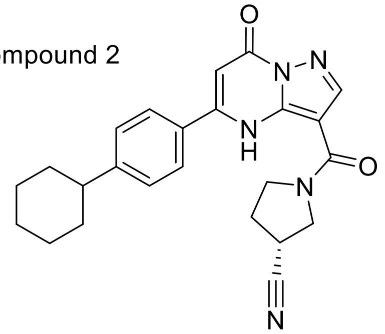

Compound 3

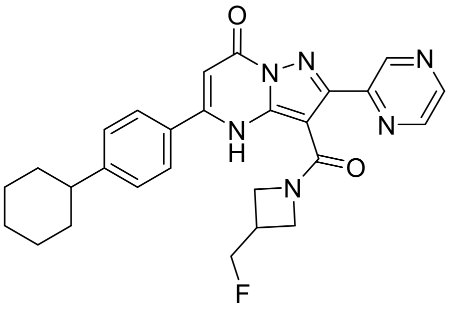

Compound 4

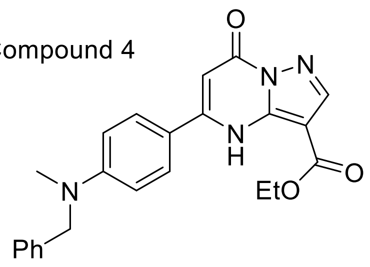

Supplement: Supplementary file 3 — Chemistry and compound note. [file 43018_2023_577_MOESM3_ESM.pdf]
