# Supplementary figures and images for: An allosteric pan-TEAD inhibitor blocks oncogenic YAP/TAZ signaling and overcomes KRAS G12C inhibitor resistance
Source: Nat Cancer. 2023 Jun 5;4(6):812–28. doi: 10.1038/s43018-023-00577-0 (PMC10293011; doi:10.1038/s43018-023-00577-0)

Source data: Uncropped western blots for Figure 1f

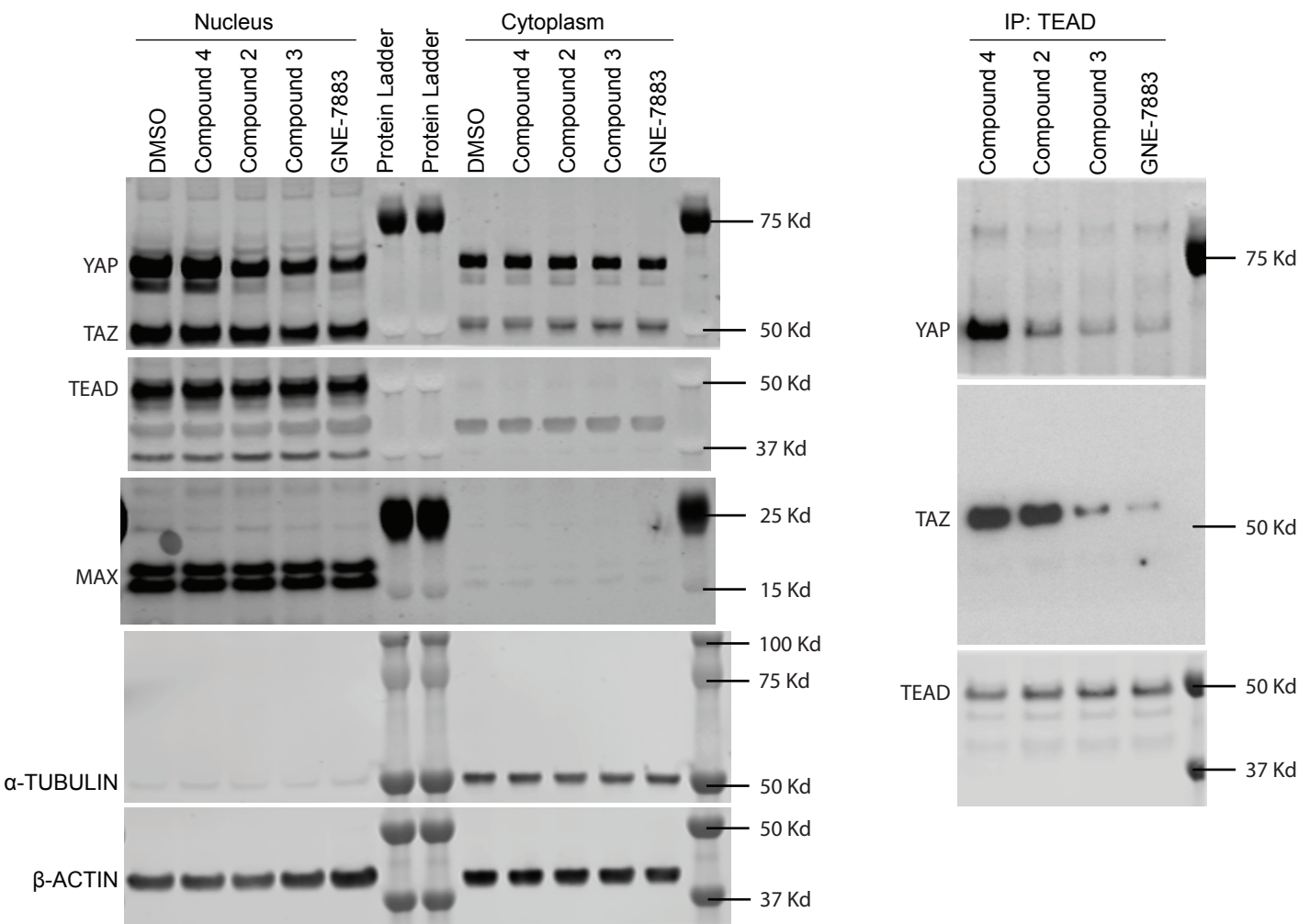

Supplement: Source Data Fig. 1 — Uncropped western blots. [file 43018_2023_577_MOESM4_ESM.pdf]

Source data: Uncropped western blots for Figure 4b

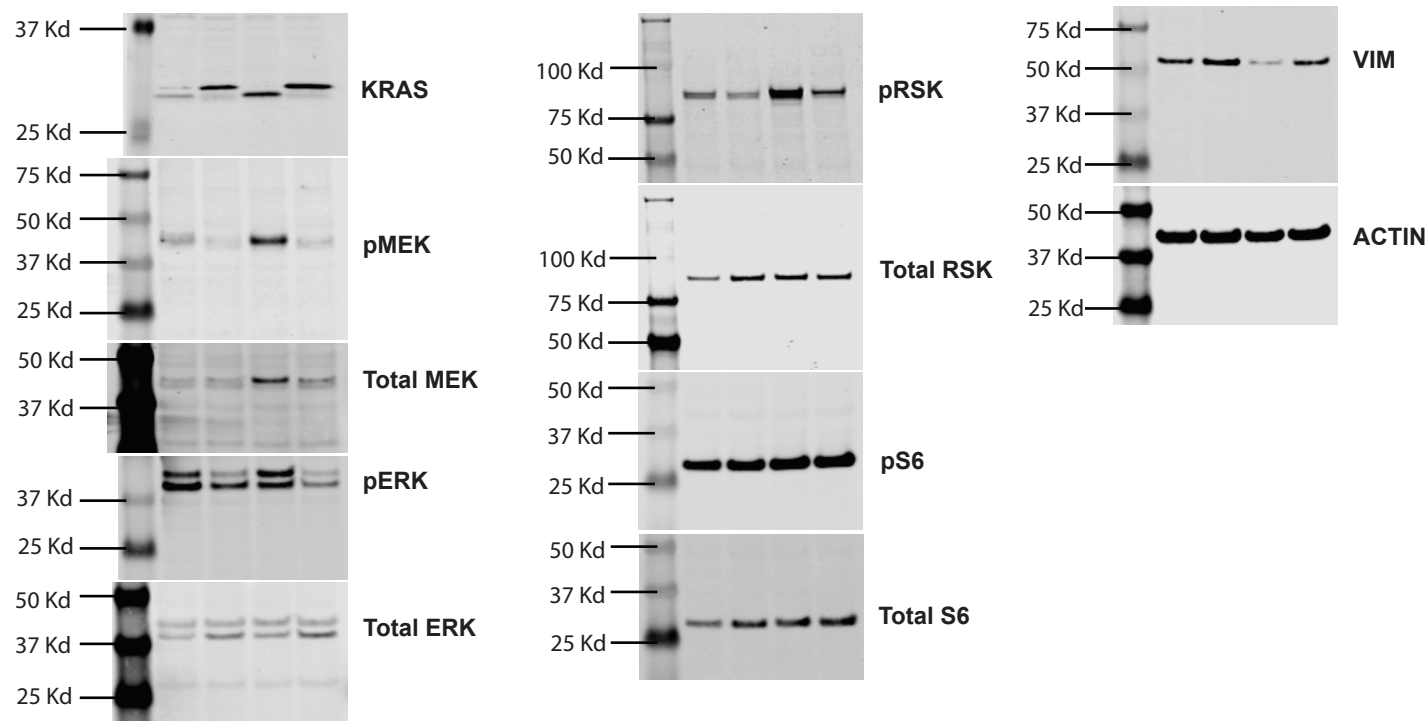

Supplement: Source Data Fig. 4 — Uncropped western blots. [file 43018_2023_577_MOESM8_ESM.pdf]
